# The anti-apoptotic *Coxiella burnetii* effector protein AnkG is a strain specific virulence factor

**DOI:** 10.1038/s41598-020-72340-9

**Published:** 2020-09-21

**Authors:** Walter Schäfer, Teresa Schmidt, Arne Cordsmeier, Vítor Borges, Paul A. Beare, Julian Pechstein, Jan Schulze-Luehrmann, Jonas Holzinger, Nicole Wagner, Christian Berens, Carsten Heydel, João Paulo Gomes, Anja Lührmann

**Affiliations:** 1 Mikrobiologisches Institut – Klinische Mikrobiologie, Immunologie und Hygiene, Universitätsklinikum Erlangen, Friedrich-Alexander-Universität Erlangen-Nürnberg, Wasserturmstraße 3/5, 91054 Erlangen, Germany; 2 Department of Infectious Diseases, National Institute of Health, Lisbon, Portugal; 3 Coxiella Pathogenesis Section, Laboratory of Bacteriology, Rocky Mountain Laboratories, National Institute of Allergy and Infectious Diseases, National Institutes of Health, Hamilton, MT USA; 4 Institut für Molekulare Pathogenese, Friedrich-Loeffler-Institut, 07743 Jena, Germany; 5 Institut für Hygiene und Infektionskrankheiten der Tiere, Justus Liebig Universität Gießen, Frankfurter Straße 85-89, 35392 Gießen, Germany

**Keywords:** Cell death and immune response, Cellular microbiology

## Abstract

The ability to inhibit host cell apoptosis is important for the intracellular replication of the obligate intracellular pathogen *Coxiella burnetii*, as it allows the completion of the lengthy bacterial replication cycle. Effector proteins injected into the host cell by the *C. burnetii* type IVB secretion system (T4BSS) are required for the inhibition of host cell apoptosis. AnkG is one of these anti-apoptotic effector proteins. The inhibitory effect of AnkG requires its nuclear localization, which depends on p32-dependent intracellular trafficking and importin-α1-mediated nuclear entry of AnkG. Here, we compared the sequences of *ankG* from 37 *C. burnetii* isolates and classified them in three groups based on the predicted protein size. The comparison of the three different groups allowed us to identify the first 28 amino acids as essential and sufficient for the anti-apoptotic activity of AnkG. Importantly, only the full-length protein from the first group is a *bona fide* effector protein injected into host cells during infection and has anti-apoptotic activity. Finally, using the *Galleria mellonella* infection model, we observed that AnkG from the first group has the ability to attenuate pathology during in vivo infection, as it allows survival of the larvae despite bacterial replication.

## Introduction

*Coxiella burnetii* is a zoonotic Gram-negative pathogen with an obligate intracellular lifestyle. The bacteria cause Q fever in humans. The acute form of Q fever is mainly characterized by a self-limiting flu-like illness and rarely by an interstitial pneumonia or hepatitis^1^. Acute Q fever is treatable with doxycycline, but many patients might also develop the post-Q fever fatigue syndrome^2^, which can last for up to 10 years^3^. At present, there is no evidence-based recommendation for treatment of post-Q fever fatigue syndrome. In addition, the infection may progress to chronic Q fever, which mainly manifests as a potentially fatal endocarditis^1^. Chronic Q fever develops months or years after infection, suggesting that *C. burnetii* persists silently within the host before massive bacterial replication leads to chronic Q fever. To control the infection, patients have to be treated for at least 18 months with doxycycline in combination with chloroquine^4^. Thus, a much more effective treatment for chronic Q fever would be highly desirable.

In general, humans become infected by inhaling *C. burnetii-*containing aerosols. Monocytes and macrophages are the primary target cells that take up *C. burnetii* by α_v_β_3_ integrin-mediated phagocytosis^5,6^. The invasion into non-phagocytic cells seems to be at least partially dependent on the outer membrane protein A (OmpA) from *C. burnetii*^7^. After uptake, the *C. burnetii*-containing vacuole (CCV) matures by consecutive fusion events to a phagolysosome-like compartment^8–10^. Usually, phagocytosis of bacterial microorganisms leads to their destruction in the phagolysosome^11^. However, *C. burnetii* survives within the phagolysosome-like environment and requires these conditions for the generation of its progeny^12^. Cellular apoptosis is also important in the defense against intracellular pathogens^13^. As this is an anti-inflammatory mechanism it eliminates infected cells without inducing inflammation and tissue destruction^14^. Yet, *C. burnetii* efficiently inhibits host cell apoptosis^15,16^. Thus, inhibition of apoptosis and withstanding phagosomal maturation are thought to be important evasion strategies of *C. burnetii*^17^. These processes are dependent on the T4BSS^18,19^, which injects effector proteins into the host cell cytosol to manipulate the host cell for the benefit of the pathogen^20^. Roughly 150 putative *C. burnetii* effector proteins have been identified; however, functions have been assigned to only a few of them^21–29^. Anti-apoptotic activity was shown for the three effector proteins AnkG, CaeA and CaeB^23,26,30–33^. Their exact mode(s) of interference with the host cell apoptotic machinery has not been determined. AnkG is the best-characterized *C. burnetii* effector protein with anti-apoptotic activity^32,33^. It is a 38.6 kDa protein and contains five predicted ankyrin-repeats, spanning from amino acids 15 to 80 and from 90 to 194. Translocation of AnkG into the host cell was suggested to be independent of the chaperone IcmS^34^. Inside the host cell, AnkG associates mainly with mitochondria and, after stress induction, localizes in the host cell nucleus^32^. Only nuclear-localized AnkG is able to prevent pathogen-induced host cell death^26,32^. Migration to and uptake into the nucleus depend on binding to the host cell proteins p32 and importin-α1, respectively^32,33^. Here, we aimed to increase our knowledge of the molecular requirements of AnkG activity, using dissimilarities found in the AnkG sequence from 37 *C. burnetii* strains.

## Results

### *C. burnetii* isolates reveal genetic variability of AnkG

Recently, the sequences of the *C. burnetii* anti-apoptotic effector protein CaeA from 25 different isolates were compared, which allowed the identification of a functional domain with anti-apoptotic activity^31^. In this study, we analyzed the *ankG* sequences from 37 *C. burnetii* isolates, including the 25 isolates from the CaeA study (Table 1) in order to learn more about the link between the *ankG* genetic background and protein function. Importantly, most of the isolates were collected from ruminants with an abnormal birthing situation, suggesting that these isolates were associated with pathology. However, if *C. burnetii* either caused or was involved with the pathology is unknown.

**Table 1:** *Coxiella burnetii* isolates used in this study.

Isolate	Host	Material	Clinic	Origin	Year
Nine Mile II RSA 439	–	–	–	Montana, USA	1985
CS-P 31	Unknown	Unknown	Unknown	unknown	unknown
Boren	Cattle	Milk	Unknown	unknown	unknown
Z 2775/90	Cattle	Afterbirth	Unknown	Rabenau, Germany	1990
Z 3027/91	Cattle		Unknown	unknown	1991
Z 3205/91a	Cattle	Afterbirth	Abortion	Rostock, Germany	1991
Z 410/94	Cattle	Afterbirth	Abortion	unknown	1994
Z 163/95	Cattle	Afterbirth	Unknown	unknown	1995
Z 66/96	Goat	Fetus	Abortion	Driedorf, Germany	1996
Z 232–3/02	Cattle		Unknown	Bobingen, Germany	2002
Z 488/94	Cattle	Afterbirth	Abortion	Lauterbach, Germany	1994
Z 502/99	Cattle		Unknown	unknown	1999
Z 3351/92	Cattle		Abortion	Germany	1992
Z 3567/92	Cattle	Afterbirth, fetus	Abortion	Lower Saxony, Germany	1992
Z 3568/92	Cattle	Fetus	Abortion	Lower Saxony, Germany	1992
Nine Mile I RSA 493	Tick	Tick	–	Montana, USA	1935
Z 3055/91	Sheep	Vaginal swab	Unknown	Haiger, Germany	1991
Henzerling RSA 331	Human	Blood	Acute Q fever	Italy	1945
71–3	Goat	Afterbirth	Unknown	Altkirchen, Germany	2010
98/2	Cattle	Organ material	Unknown	Altenberga, Germany	2010
Soyta/6/65	Cattle	Unknown	Unknown	Switzerland	1965
19/34	Goat	afterbirth, fetus	Abortion	Bissingen, Germany	2010
23/2	Sheep	Afterbirth, fetus	Abortion	Jork, Germany	2011
W-3	Fallow deer	Afterbirth	Abortion	Gäufelden, Germany	1997
W-4	Sheep	Afterbirth	Abortion	Aidlingen, Germany	1996
Z 104/94	Sheep	Afterbirth	Unknown	Dillenburg, Germany	1994
Z 346/99	Sheep	Afterbirth	Abortion	Sarstedt, Germany	1999
Z 3464/92	Goat	Afterbirth	Abortion	Pohlheim, Germany	1992
Z 3468–5/92	Sheep		Unknown	Lower Saxony, Germany	1992
Z 3478/92	Sheep	Fetus	Abortion	Pohlheim, Germany	1992
Z 4485/93	Sheep	Afterbirth	Abortion	Dillenburg, Germany	1993
Andelfingen/23/2–65	Cattle	Unknown	Unknown	Switzerland	1965
Frankfurt	Cattle	Milk	Unknown	Hesse, Germany	1982
F-3/UR.CB.L.IE. 15	Human	Mitral valve	Endocarditis	Lyon, France	1978
F-4/UR.CB.M.IE. 24	Human	Blood, valve	Endocarditis	Marseille, France	1978
Z 3574–1/92	Sheep	Milk	Unknown	Berlin, Germany	1992
Namibia	Goat	Unknown	Unknown	Windhoek, Namibia	1991

**Figure 1:**
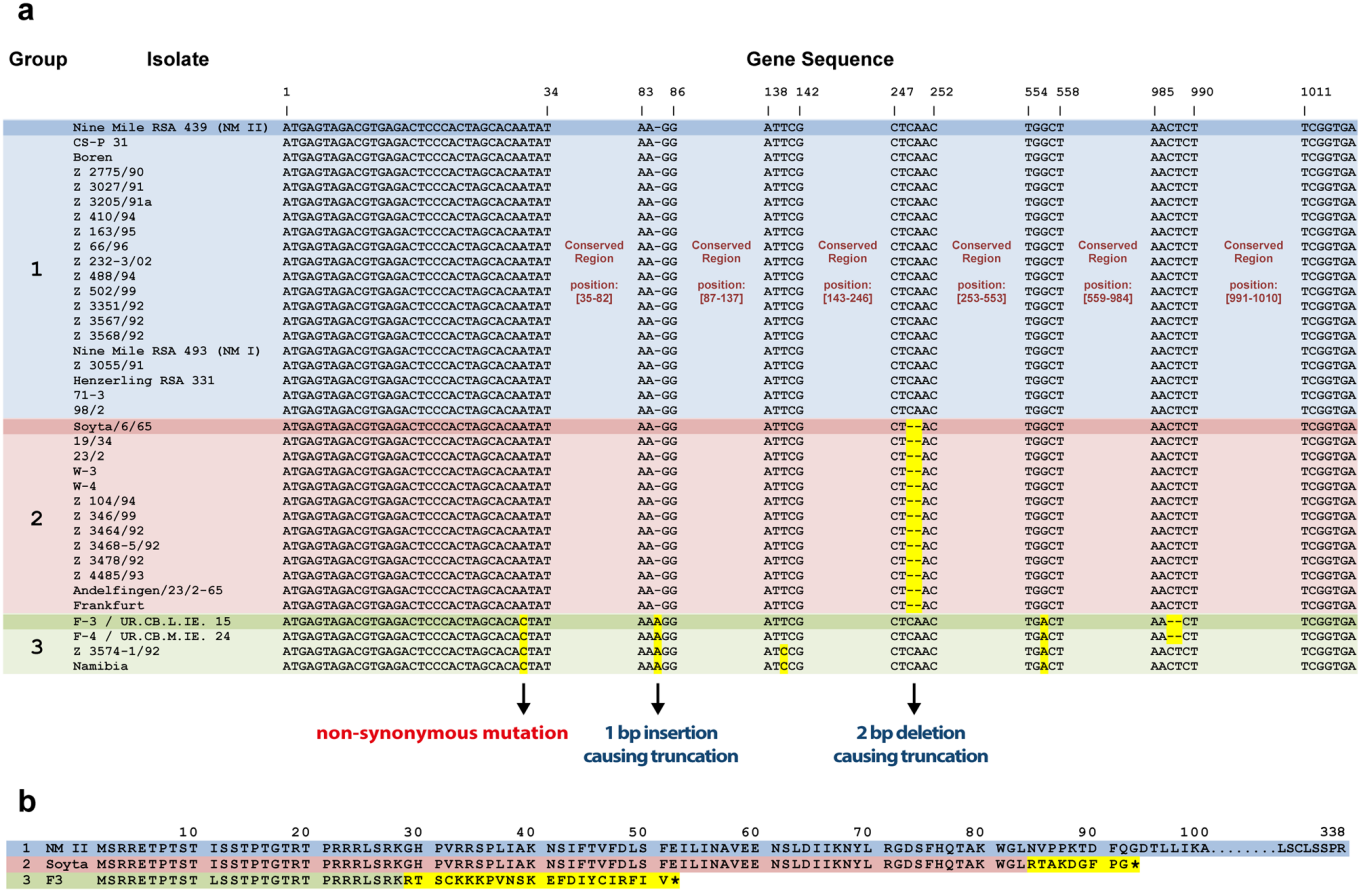
Genomic diversity of *ankG*. (**a**) The *ankG* alignment defines three groups, which are highlighted by colors (blue, red and green). Single base insertions, deletions and nucleotide substitutions are indicated either by dashes or highlighted in yellow. (**b**) AnkG amino acid sequences of three *C. burnetii* isolates –each representatives for its group. The first group contains the reference strain Nine Mile II (AnkG_NM_) and nineteen additional strains expressing a 338 amino acid protein (blue). The second group includes thirteen isolates and is represented by *C. burnetii* Soyta (AnkG_Soyta_) expressing a 92 amino acid protein. AnkG_Soyta_ is identical in the first 83 N-terminal amino acids (red), but harbors 9 different amino acids at the C-terminus (yellow) compared to AnkG_NM_. The third group contains four isolates and is represented by *C. burnetii* strain F3 (AnkG_F3_) expressing a 51 amino acid protein. AnkG_F3_ has an amino acid exchange at position 11 (isoleucine to leucine) and is otherwise identical to AnkG_NM_ in the first 28 N-terminal amino acids (green), but contains 23 different amino acids at the C-terminus (yellow). The asterisk (*) at the C-terminus of the amino acid sequence representing the second and third group indicates premature truncation.

By comparing the 37 different *ankG* sequences, we observed a high degree of similarity at the nucleotide level (Fig. 1a). However, some strains contained base pair deletions or insertions, leading to frame shifts and, consequently, the introduction of a premature stop codon. Based on this in silico analysis the strains were classified into three groups (Fig. 1b).

The reference strain Nine Mile (NM) and nineteen further isolates express the full-length 338 amino acid protein of 38.6 kDa and belong to the first group. Importantly, the sequence of *ankG* is identical in NM phase I and NM phase II (Fig. 1a). The thirteen isolates of the second group contain a two base pair deletion directly after amino acid 83, leading to a 92 amino acid protein of 10.4 kDa. Thus, AnkG from this group (AnkG_Soyta_) is identical to the first group in amino acids 1 to 83, but substantially differs afterwards in both size and protein sequence.

Only four isolates belong to the third group, which differs from the groups one and two in an exchange of isoleucine into leucine at position 11. This mutation is also present in the Dugway strain. It leads to a slightly, but significantly, better protection from staurosporine-induced apoptosis^33^. In addition, AnkG from the third group contains a base pair insertion at the codon for amino acid 29, leading to a premature stop at amino acid codon 51 and a protein of 6 kDa. AnkG from this group (AnkG_F3_) is identical to AnkG_NM_ in the amino acids 1–28, but different in the amino acids 29–51.

### The AnkG variants from the three groups respond differently to apoptosis induction

**Figure 2:**
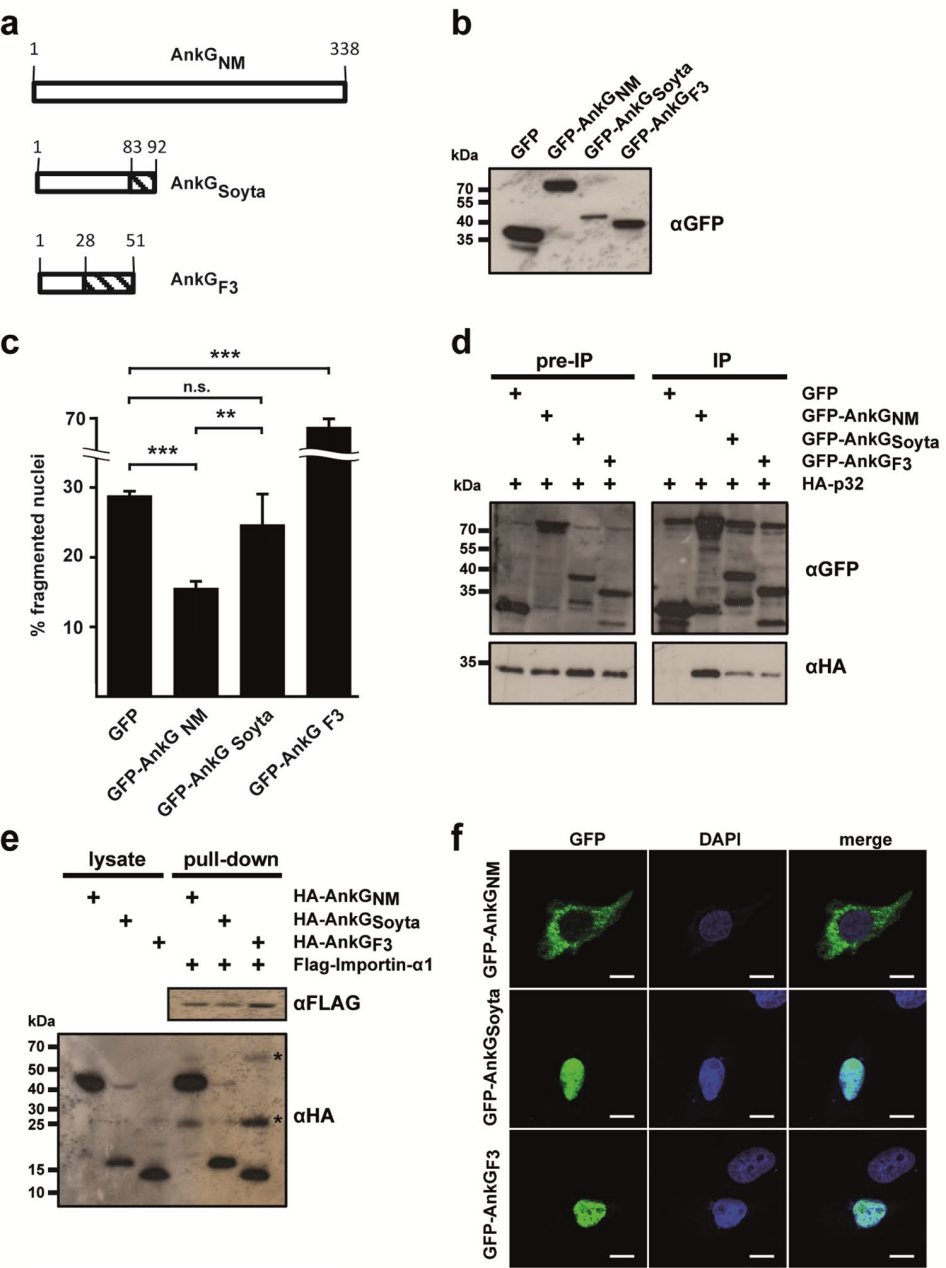
The three AnkG groups display different anti-apoptotic activities. (**a**) Schematic representation of the AnkG variants used in this study. Amino acids are shown above each of the schematic diagrams. Striped area represents altered amino acids as compared to the reference AnkG_NM_. (**b**) CHO-FcR cells were transiently transfected with GFP, GFP-AnkG_NM_, GFP-AnkG_Soyta_ or GFP-AnkG_F3_. Proteins were separated by SDS-PAGE, transferred to a PVDF membrane and probed with an antibody against GFP. Depicted is one representative immunoblot of three independently performed experiments with similar results. (**c**) CHO-FcR cells expressing GFP, GFP-AnkG_NM_, GFP-AnkG_Soyta_ or GFP-AnkG_F3_ were treated with staurosporine for 4 h. The cells were fixed, permeabilized and the nuclei were stained with DAPI. The nuclear morphology of at least 100 GFP-expressing cells was scored in three independent experiments. Error bars indicate ± SD. **p < 0.01; ***p < 0.001, n.s. not significant. (**d**) HEK293 cells were co-transfected with plasmids encoding HA-tagged p32, GFP-tagged AnkG_NM_, AnkG_Soyta_ and AnkG_F3_. The proteins were precipitated from the cell lysates using a GFP-trap. Immunoblot analysis was used to detect p32 (anti-HA) and AnkG-protein variants (anti-GFP) in the lysates (pre-IP) and in the precipitates (IP). Shown is a representative experiment out of three independent experiments with similar results. (**e**) Cell lysates from HEK293 cells transiently expressing HA-tagged AnkG_NM_, AnkG_Soyta_ and AnkG_F3_ were incubated with Flag-tagged importin-α1 bound to an anti-Flag M2 affinity gel. Cell lysates and pull-down fractions were subjected to immunoblot analysis using anti-HA and anti-Flag antibodies. Shown is a representative experiment from three independent experiments with similar results. *Unspecific band. (**f**) Representative immunofluorescence micrographs show CHO-FcR cells transiently expressing GFP-AnkG_NM_, GFP-AnkG_Soyta_ or GFP-AnkG_F3_ (all green) stained with DAPI to visualize the nuclei (blue). Scale bars, 10 µm.

We previously demonstrated that the amino acids 1–69 of AnkG_NM_ are required for anti-apoptotic activity^26^. Therefore, we analyzed whether AnkG_Soyta_ (member of the second group), which contains all 69 amino acids of the first group, or AnkG_F3_ (member of the third group), where only the first 28 amino acids, except for amino acid 11, are identical to AnkG_NM_, would affect host cell survival (Fig. 2a). We ectopically expressed GFP or GFP-AnkG variants transiently in CHO cells. First, we demonstrated stable expression by immunoblot analysis (Fig. 2b). Next, we quantified the percentage of fragmented nuclei as readout for apoptosis after treatment with the apoptosis-inducer staurosporine. Our results demonstrated that AnkG of the first group (AnkG_NM_) inhibited apoptosis, AnkG of the second group (AnkG_Soyta_) did not affect apoptosis induction, and AnkG from the third group (AnkG_F3_) displayed apoptosis-promoting activity (Fig. 2c). Importantly, without staurosporine-induction, AnkG_F3_ did not display any pro-apoptotic activity (data not shown). As the anti-apoptotic activity of AnkG_NM_ requires nuclear localization, which is mediated by binding to p32- and importin-α1^32,33^, we analyzed the interaction of the AnkG variants with the host cell proteins p32 and importin-α1. We hypothesized that AnkG_Soyta_ and AnkG_F3_ bind both host cell proteins, as the binding of AnkG_NM_ to importin-α1 was mapped to the amino acids surrounding residue 11, while the amino acids neighbouring residues 22 and 23 had been identified as the binding region for the interaction with p32^32,33^. Indeed, as shown in Figs. 2d and 2e, all three AnkG variants were able to bind to p32 and importin-α1. The interaction of the HA-AnkG variant with Flag-tagged importin-α1 is not mediated indirectly by the anti-FLAG beads, as shown by us before^33^. Next, we analyzed the subcellular localization of the AnkG variants, as nuclear localization of AnkG_NM_ was demonstrated to be essential for its anti-apoptotic activity^32^. AnkG_NM_ displays vesicular staining with close association with host cell mitochondria in healthy cells. After cellular stress, AnkG_NM_ migrates to the host cell nucleus. In contrast, AnkG_NM 1–69_ was present within the host cell nucleus under all conditions^32^. Thus, we hypothesized that without cell stress induction the AnkG variants from the second and third groups might also localize to the nucleus, which was, in fact, the case (Fig. 2f). Taken together, although AnkG_Soyta_ bound to p32 and importin-α1 and was translocated to the nucleus, it was unable to inhibit apoptosis. AnkG_F3_ was also found in the nucleus and bound to p32 and importin-α1, but displayed staurosporine-dependent pro-apoptotic activity in contrast to the anti-apoptotic activity of AnkG_NM_.

### Characterization of AnkG_Soyta_ activity

**Figure 3:**
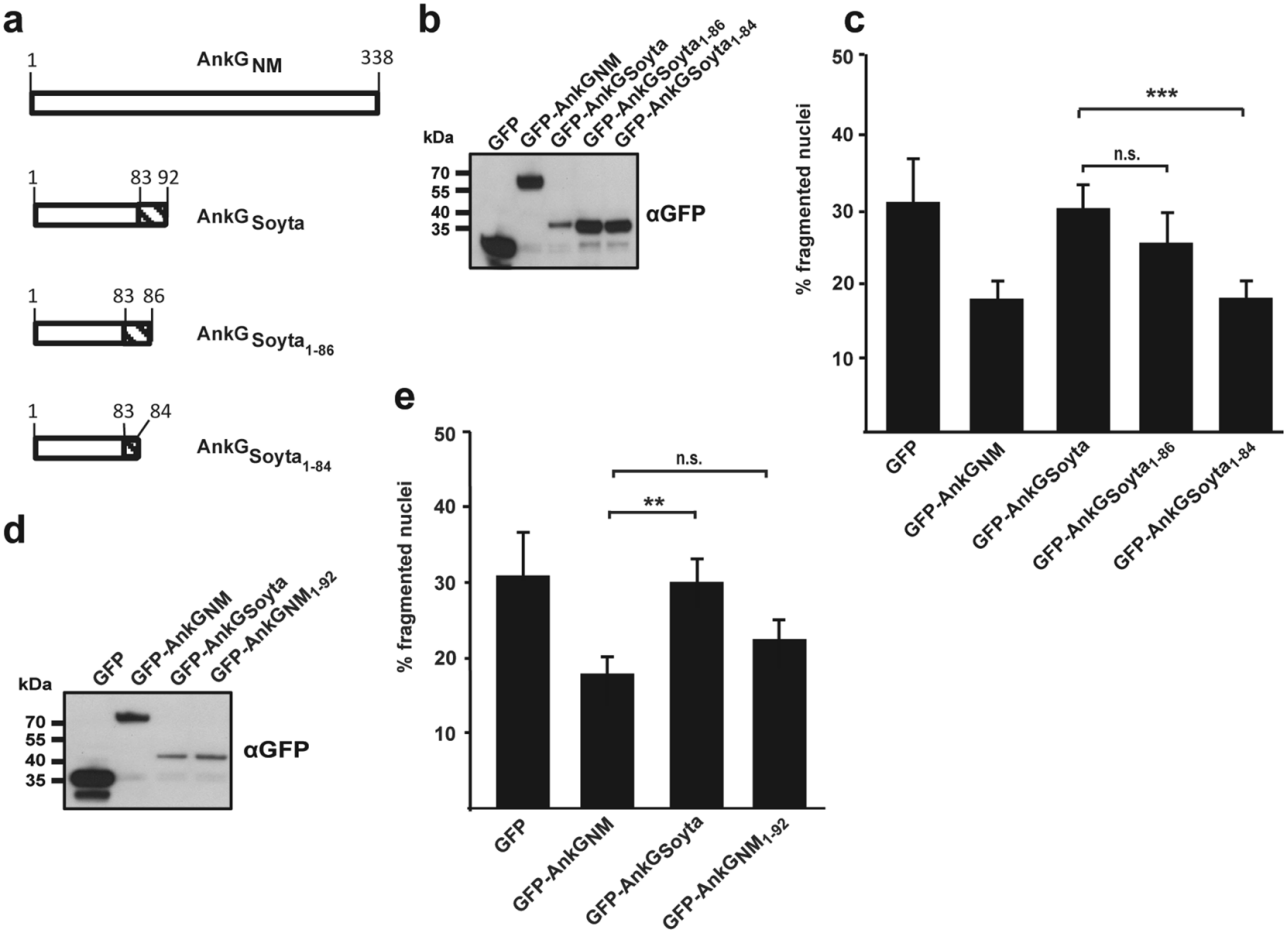
Characterization of AnkG_Soyta_-mediated apoptosis modification. (**a**) Schematic representation of the AnkG_NM_ and AnkG_Soyta_ truncations used. Amino acids are shown above each of the schematic diagrams. The striped area represents altered amino acids as compared to the reference sequence AnkG_NM_. (**b**–**e**) CHO-FcR cells were transiently transfected with GFP or with the GFP-tagged AnkG variants indicated. (**b**,**d**) Proteins were separated by SDS-PAGE, transferred to a PVDF membrane and probed with an antibody against GFP. Depicted is one representative immunoblot of three independently performed experiments with similar results. (**c**,**e**) CHO-FcR cells expressing either GFP or the indicated GFP-tagged AnkG variants were treated with staurosporine for 4 h. The cells were fixed, permeabilized and the nuclei stained with DAPI. The nuclear morphology of at least 100 GFP-expressing cells was scored in three independent experiments. Error bars indicate ± SD. **p < 0.01; ***p < 0.001, n.s. not significant.

AnkG_Soyta_ represents the second group and forms a 10.4 kDa protein. While the first 83 amino acids are identical to AnkG_NM_, the residues 84 – 92 differ. Although AnkG_Soyta_ bound to p32 and importin-α1 and displayed nuclear localization, it was unable to inhibit apoptosis. To investigate the underlying reason(s), we first constructed truncations of AnkG_Soyta_ by stepwise removal of amino acids from its C-terminus (Fig. 3a). We ectopically expressed GFP or GFP-AnkG_Soyta_ variants transiently in CHO cells and demonstrated stable expression by immunoblot analysis (Fig. 3b). Next, we measured apoptosis after stimulation with staurosporine by quantifying nuclear fragmentation, which was visualized by DAPI staining. As shown in Fig. 3c, stepwise removal of amino acids from the C-terminal region in AnkG_Soyta_ restored its anti-apoptotic activity. This suggests that the C-terminal region in AnkG_Soyta_ disturbs the activity of the protein. We hypothesized that this might have been caused by the length of the protein. To address this experimentally, we constructed a truncated AnkG_NM 1–92_ to clarify whether the length of the protein might be the cause for the lost activity of AnkG_Soyta_, which also contains 92 amino acids. As shown in Fig. 3d, all constructs were ectopically expressed following transient transfection of CHO cells. AnkG_NM 1–92_ inhibited staurosporine-induced apoptosis as efficiently as full-length AnkG (Fig. 3e), indicating that the reduced length is not the cause for the missing anti-apoptotic activity of AnkG_Soyta_. These results suggest that the amino acids 84–92 of AnkG_Soyta_ might influence the folding or intra- or intermolecular binding capacity of the protein.

### Characterization of AnkG_F3_ activity

**Figure 4:**
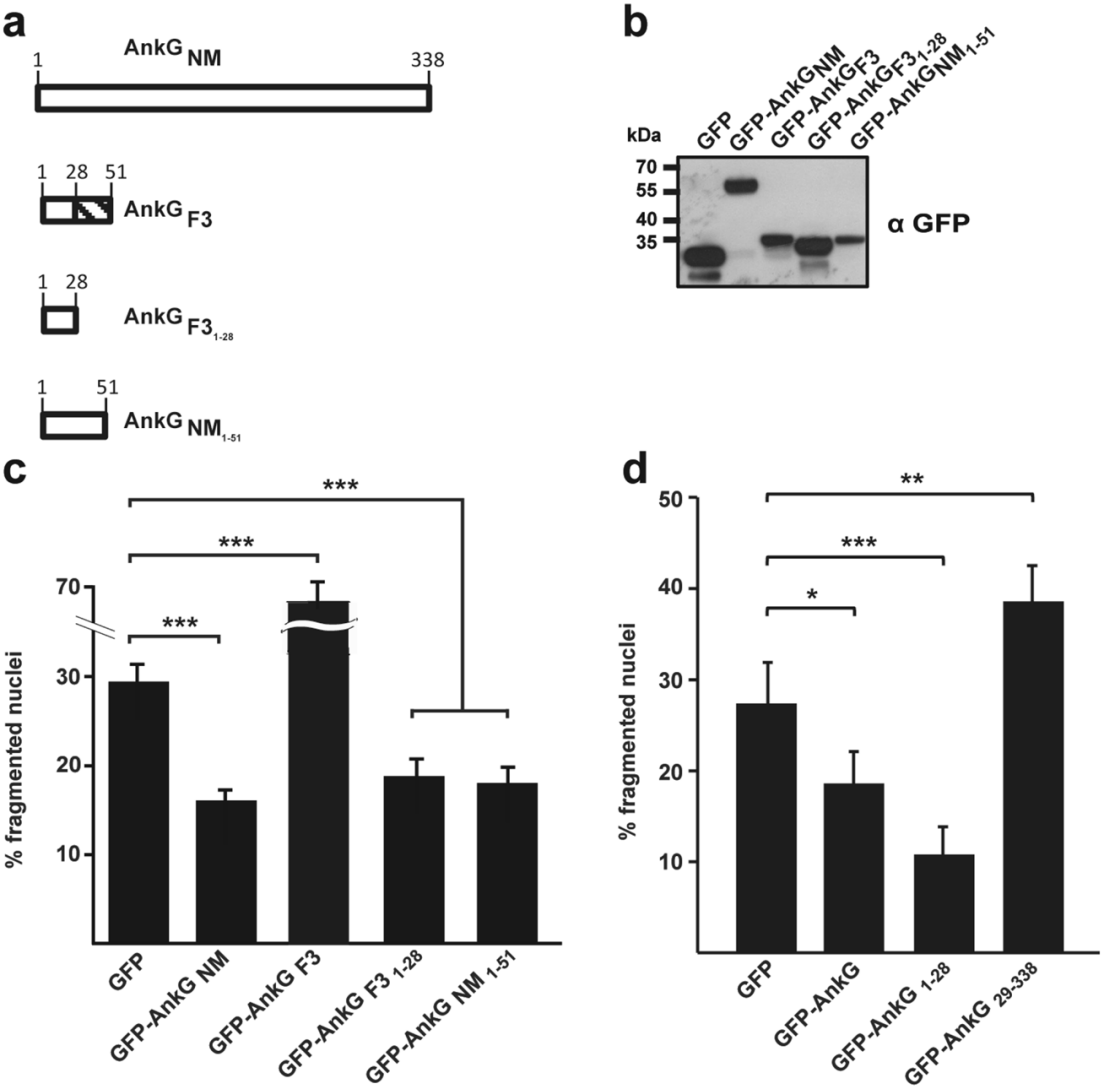
Characterization of AnkG_F3_-mediated apoptosis induction. (**a**) Schematic representation of the AnkG variants used. Amino acids are shown above each of the schematic diagrams. The striped area represents altered amino acids as compared to the reference AnkG_NM_ sequence. (**b**) CHO-FcR cells were transiently transfected with GFP or with the GFP-tagged AnkG variants indicated. Proteins were separated by SDS-PAGE, transferred to a PVDF membrane and probed with an antibody against GFP. Depicted is one representative immunoblot of three independently performed experiments with similar results. (**c**, **d**) CHO-FcR cells expressing GFP or the indicated GFP-tagged AnkG variants or truncations were treated with staurosporine for 4 h. The cells were fixed, permeabilized and the nuclei were stained with DAPI. The nuclear morphology of at least 100 GFP-expressing cells was scored in three independent experiments. Error bars indicate ± SD. *p < 0.05; **p < 0.01, ***p < 0.001.

AnkG_F3_ represents the third group and forms a 6 kDa protein. While the first 28 amino acids, except for amino acid 11, are identical to AnkG_NM_, the amino acids 29 – 51 are different. AnkG_F3_ bound to p32 and importin-α1 and translocated into the nucleus, but it showed pro-apoptotic activity after apoptosis-induction. To shed light on the underlying reason, we created different AnkG truncations (Fig. 4a). We constructed a truncated variant AnkG_F3 1–28_ to clarify whether the first 28 amino acids, which are identical between AnkG_F3_ and AnkG_NM_, are sufficient for anti-apoptotic activity. Additionally, we constructed AnkG_NM 1–51_ to clarify whether the length of the protein might result in a change of its activity. First, we ectopically expressed GFP or the GFP-AnkG variants transiently in CHO cells and demonstrated stable expression by immunoblot analysis (Fig. 4b). As shown in Fig. 4c, while GFP-AnkG_F3_ significantly increases staurosporine-induced apoptosis, GFP-AnkG_F3 1–28_ inhibited apoptosis similarly to GFP-AnkG_NM_. This demonstrates that AnkG_F3_ contains the anti-apoptotic domain, which comprises the first 28 amino acids. Expression of GFP-AnkG_NM 1–51_ also resulted in inhibition of apoptosis, indicating that the length of AnkG_F3_, which contains 51 amino acids, cannot explain its pro-apoptotic activity. These findings indicate that the amino acids 29–51 of AnkG_F3_ negatively affect the anti-apoptotic activity of the N-terminal region.

To determine whether the amino acids 1–28 are necessary and sufficient for the anti-apoptotic activity, we ectopically expressed GFP, GFP-AnkG_NM_, GFP-AnkG_NM 1–28_ or GFP-AnkG_NM 29–338_ transiently in CHO cells and determined cell death after staurosporine treatment. The expression of GFP-AnkG_NM 1–28_ significantly inhibited staurosporine-induced apoptosis. In contrast, the expression of GFP-AnkG_NM 29–338_ did not protect the cell from apoptosis (Fig. 4d). These data suggest that the amino acids 1–28 are necessary and sufficient for the anti-apoptotic activity of AnkG.

### Analyzing transcription of the *ankG* gene from the three different groups

**Figure 5:**
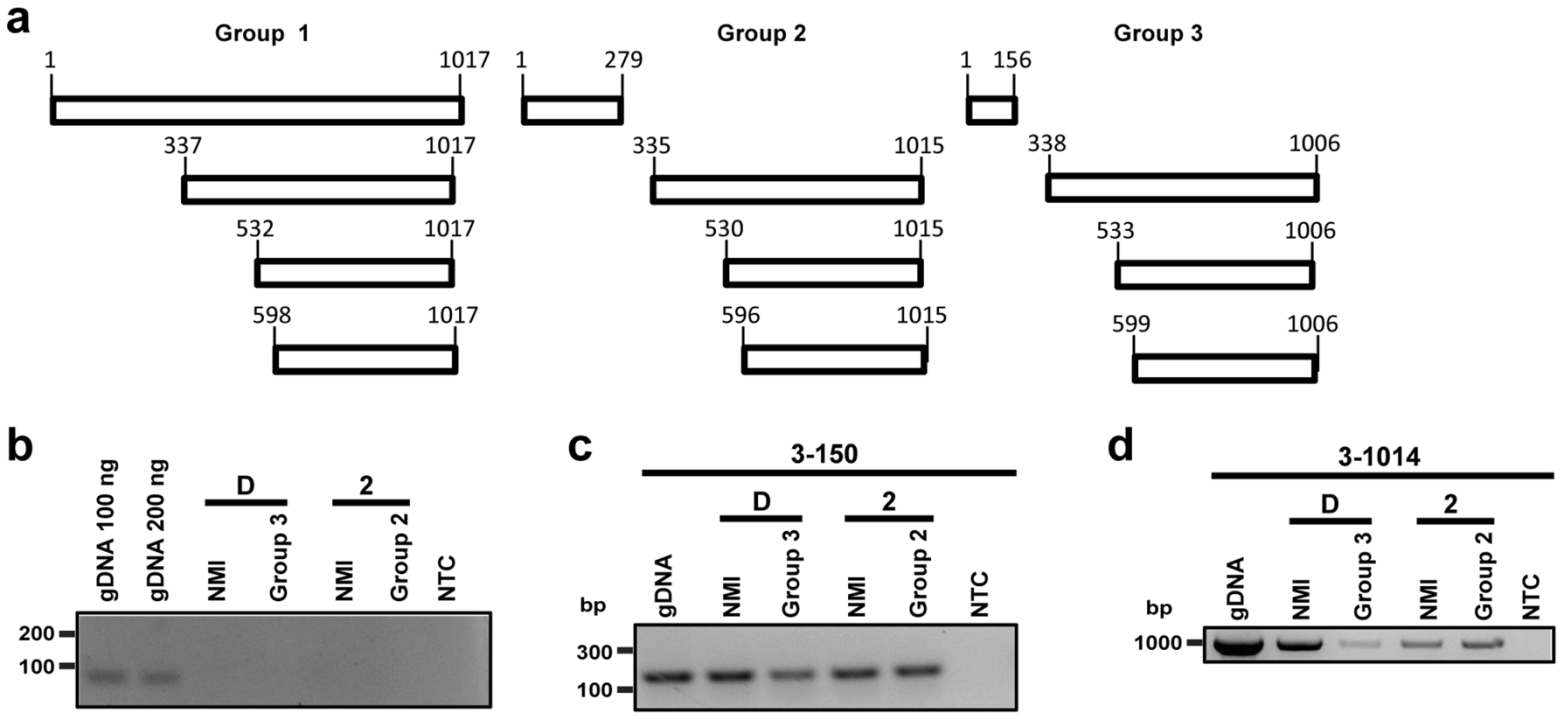
Determination of *ankG* variant transcripts. (**a**) Schematic overview of possible transcripts of the three different AnkG groups is shown. The first group is represented by NMI, the second group by 19/34, and the third group by Z3574-1/92. The numbers of the first base pair of potential internal start codons are given, which are in frame with the stop codon at base pair position 1,015–1,017 (group 1), 1,013–1,015 (group 2) or 1,004–1,006 (group 3). (**b**) DNase treated RNA was analyzed for DNA contamination using *dotA* specific primers. As positive control *C. burnetii* NMII gDNA was used in different amounts. Gel electrophoretic analysis was performed using a 2% agarose gel. (**c**, **d**) Gel electrophoresis image of the different *ankG* mRNA sections probed in the distinct *C. burnetii* strains. gDNA served as positive control, while the non-template control (NTC) contains only water. RNA was isolated from all axenically grown strains (2—grown in ACCM-2; D—grown in ACCM-D), reverse-transcribed in cDNA, and amplified by PCR. Gel electrophoretic analysis was performed using a 2% agarose gel. The cDNA was analyzed for the presence of (c) the mRNA region encoding the N-terminal part of the protein (base pairs 3–150) and (d) the full-length transcript (base pairs 3–1,014).

The *ankG* gene from the first group is transcribed and translated as a full-length protein. The *ankG* alleles from the second and third groups contain a premature stop codon leading to the translation of a 10 and 6 kDa protein, respectively. To determine whether the *ankG* variants from the second and third groups encode additional proteins containing the C-terminal translocation signal, we inspected the *ankG* sequences for the presence of additional CDS within the original full-length transcript. Indeed, *ankG* contains three further start codons, which are in-frame with the stop codon at the C-terminus. These start codons are located at slightly varying base pair positions in the three *ankG* alleles near bp 337, 532 and 598 in the respective alleles (Fig. 5a). In order to determine whether the C-terminal fragments are transcribed, we analyzed the mRNA from *C. burnetii* isolates belonging to the three different groups. As representative for the first group, we chose Nine Mile I (NMI) and for the second and third groups 19/34 and Z3574-1/92, respectively. First, we had to establish axenic growth of these isolates, as axenic media does not support growth of all *C. burnetii* strains^35^. While growth of NMI in ACCM-2 and ACCM-D has been shown, growing 19/34 and Z3574-1/92 in axenic media has never been attempted. The strain 19/34 grew similarly well in ACCM-2 as NMI. However, Z3574-1/92 was only able to grow in ACCM-D media. As *C. burnetii* has different growth kinetics in ACCM-2 and ACCM-D media^36^, which might influence transcriptional activity, we always used NMI grown in the respective media as control. Thus, we isolated RNA from 19/34 and NMI grown in ACCM-2 and RNA from Z3574-1/92 and NMI grown in ACCM-D. After treatment with DNase, we confirmed the lack of DNA contamination by performing a no reverse transcriptase control using specific primers for the *dotA* gene (Fig. 5b). Next, we reverse transcribed the mRNA into cDNA and determined whether the RNA fragment encoding the N-terminal residues of the three different *ankG* groups was transcribed. As shown in Fig. 5c, the base pairs 3–150 were transcribed in all three groups, suggesting that the 5´part of the mRNA can be transcribed and translated into the respective proteins. Next, we analyzed if the full-length *ankG* open reading frame of all three groups is transcribed into the corresponding mRNA. This is indeed the case for all three groups (Fig. 5d). Thus, the 3´ part of the *ankG* mRNA from the second and third groups is transcribed and might be translated into a protein containing the C-terminal translocation signal. Still, although these proteins might have the potential to be injected into the host cell via the T4BSS, they most likely lack anti-apoptotic activity due to missing amino acids 1–28.

### Role of AnkG_Soyta_ and AnkG_F3_ during a *C. burnetii* infection

**Figure 6:**
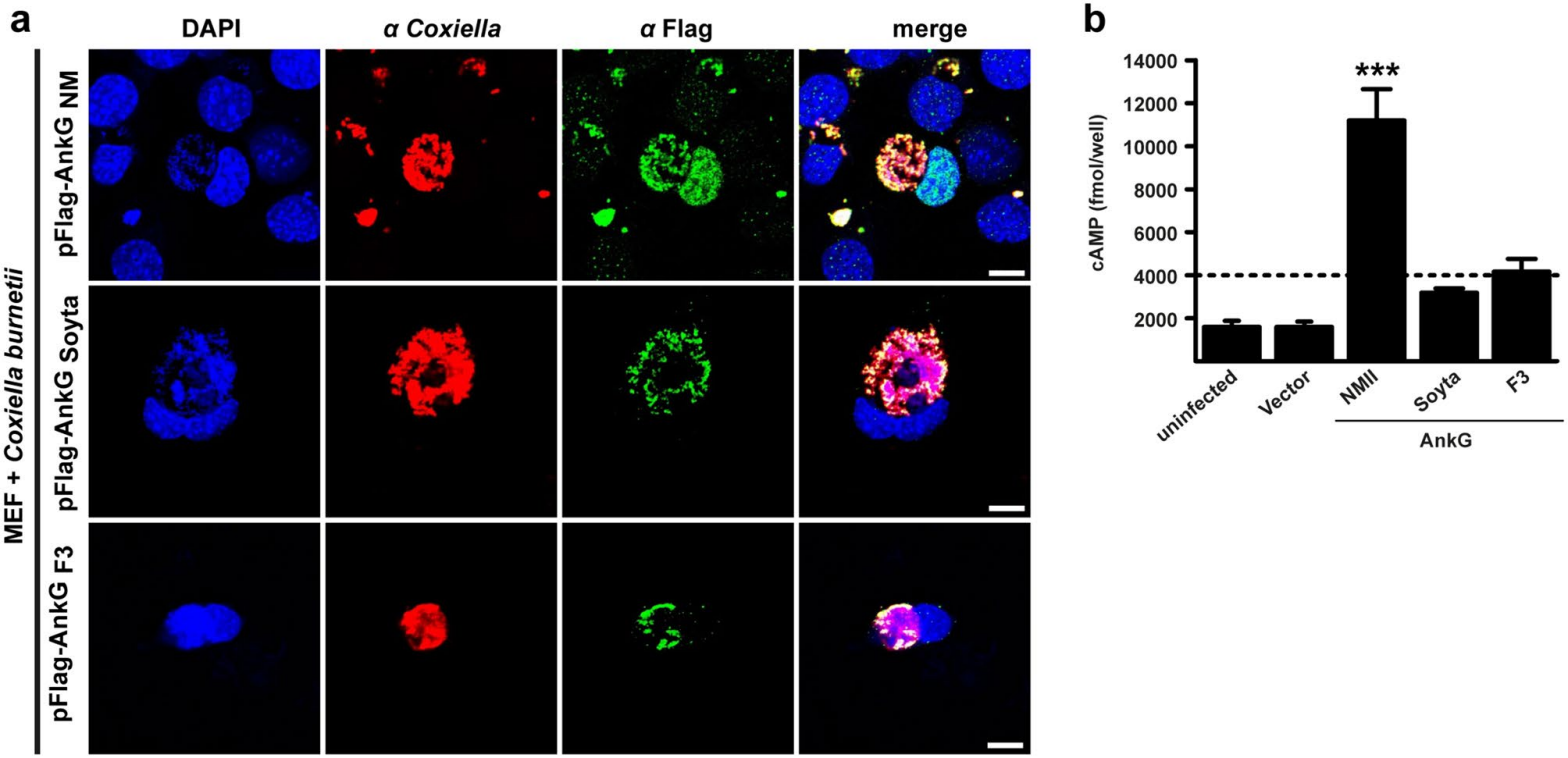
Only AnkG_NM_ is a *bona fide* T4BSS effector protein. (**a**) Representative confocal micrographs of mouse embryonic fibroblasts (MEFs) infected with *C. burnetii* expressing Flag-AnkG_NM_, Flag-AnkG_Soyta_ or Flag-AnkG_F3_. MEFs were fixed 48 h post-infection and were stained with an antibody specific for Flag (green) and *Coxiella* (red). DNA was stained with DAPI (blue). Scale bars, 10 μm. (**b**) The amount of cAMP is given in fmol/well for CHO cells either uninfected or infected for 72 h with *C. burnetii* producing Cya, Cya-AnkG_NM_, Cya-AnkG_Soyta_ or Cya-AnkG_F3_. The threshold for secretion was set to 2.5-fold the cAMP-level of the controls and is depicted as a dotted line. Shown is the mean of at least three experiments ± SD. ***p < 0.001.

During a *C. burnetii* infection AnkG_NM_ is transported into the host cell nucleus, the site of its anti-apoptotic activity^33^. While we do not fully understand the mechanism by which an effector protein is recognized for delivery through the T4BSS, we know that the C-terminal end of the effector serves as the translocation signal^37^. In contrast to AnkG_NM_, AnkG_F3_ and AnkG_Soyta_ lack the C-terminal end and should therefore not be translocated through the T4BSS. However, it was demonstrated that AnkG_NM_, AnkG_NM 1–69_ as well as AnkG_NM 70–338_ are translocated by the *Legionella pneumophila* T4BSS^26^. This indicates that AnkG_NM_ might contain two translocation signals, one in its C-terminal part and another one within the first 69 amino acids. Therefore, AnkG_Soyta_ and/or AnkG_F3_ might also be recognized as an effector protein and translocated into the host cell by the T4BSS. To determine if this is the case, we transformed *C. burnetii* with plasmids containing 3xFlag-tagged AnkG_F3_ or AnkG_Soyta_. Single *C. burnetii* pFlag-AnkG_F3_ or pFlag-AnkG_Soyta_ clones were isolated and transgene expression was analyzed (data not shown). Mouse embryonic fibroblasts (MEFs) were infected with *C. burnetii* pFlag-AnkG_NM_, *C. burnetii* pFlag-AnkG_F3_ and *C. burnetii* pFlag-AnkG_Soyta_ under inducing conditions and the localization of Flag-AnkG was analyzed by confocal imaging. As shown in Fig. 6a, Flag-AnkG_NM_ was associated with the bacteria and localized within the host cell nucleus, confirming previous results^33^. This indicates that Flag-tagged AnkG_NM_ can be translocated by *C. burnetii* into the host cell. In contrast, Flag-AnkG_Soyta_ and Flag-AnkG_F3_ were only associated with the bacteria and not present in the host cell. This indicates that neither AnkG_Soyta_ nor AnkG_F3_ are T4BSS effector proteins. To support this assumption, we fused AnkG_NM_, AnkG_Soyta_ and AnkG_F3_ to the calmodulin-dependent adenylate cyclase toxin (Cya). CHO cells were infected with *C. burnetii* producing either Cya-AnkG_NM_, Cya-AnkG_Soyta_ or Cya-AnkG_F3_ and the amount of cyclic AMP (cAMP) was measured. As Cya catalyzes the production of cAMP its amount correlates with the translocation of Cya-tagged AnkG variants into the cytoplasm. A concentration of 2.5-fold more cAMP than the negative control (Cya alone) was regarded as proof for translocation of the protein. Cya-AnkG_NM_ was translocated into the host cell cytosol (Fig. 6b), supporting previous results^26^. In contrast, Cya-AnkG_Soyta_ and Cya-AnkG_F3_ were not translocated into the host cell cytoplasm. This data supports the hypothesis that neither AnkG_Soyta_ nor AnkG_F3_ are T4BSS effector proteins.

### AnkG_NM_ attenuates pathology during *C. burnetii* infection in *Galleria mellonella*

**Figure 7:**
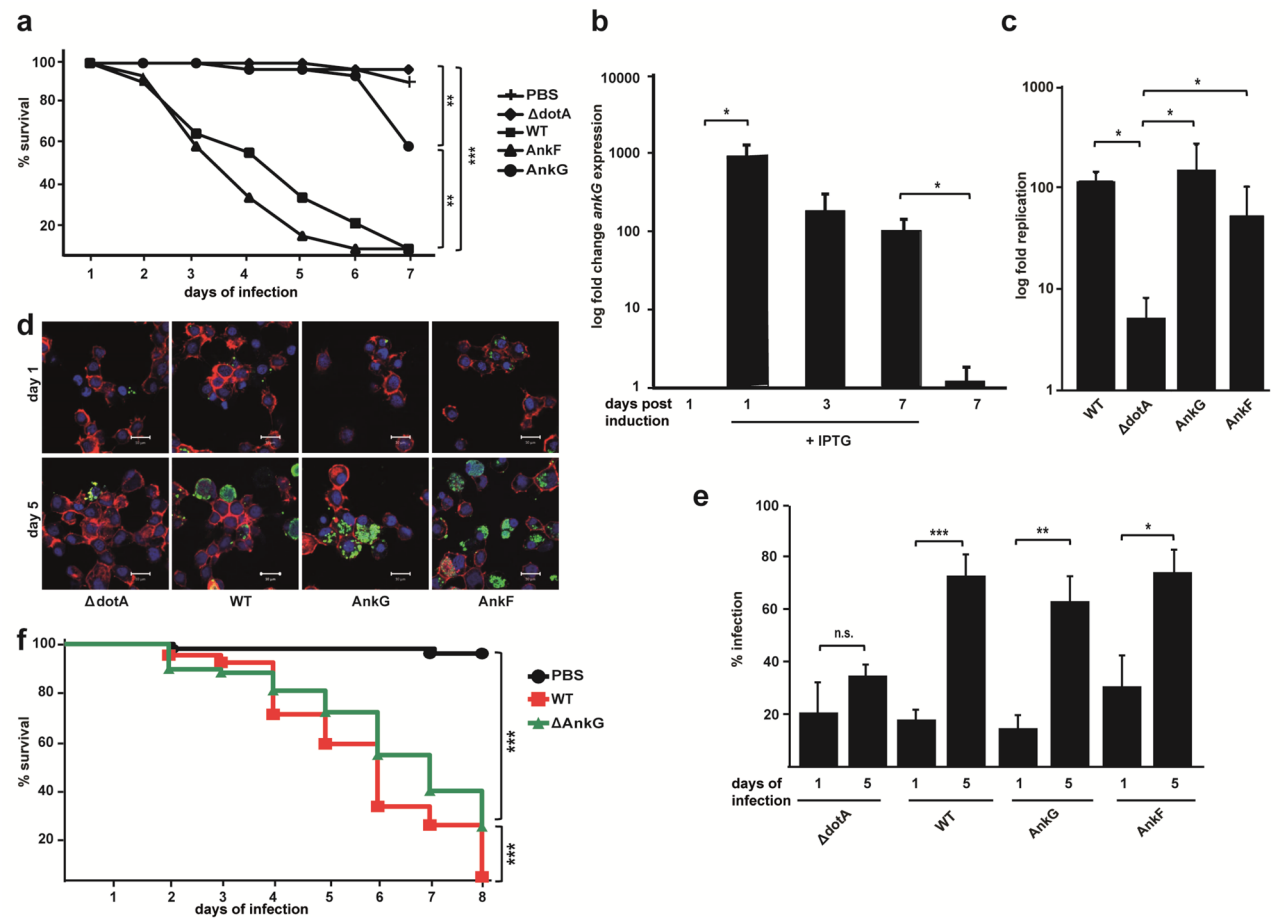
AnkG_NM_ mediates host tolerance to *C. burnetii* infection. (**a**) Survival of infected *Galleria mellonella* larvae was analyzed over a 7 day period. 10 larvae each were infected with 10^6^ bacteria (*C. burnetii* wildtype (WT), *C. burnetii ΔdotA* (ΔdotA), *C. burnetii* pFlag-AnkG (AnkG) or *C. burnetii* pFlag-AnkF (AnkF)) per larvae in 20 µl PBS or with a PBS control. Survival was controlled every 24 h. Shown is the mean of three experiments ± SD. **p < 0.01; ***p < 0.001. (**b**) AnkG level was measured by RT-PCR 1, 3 and 7 days post-induction with 2 mM IPTG and compared to the AnkG level of respective the untreated control. RNA was isolated and reverse transcribed in cDNA. Fold replication was calculated using the ΔΔCT method. Shown is the mean of three independent experiments. *p < 0.05*.* (**c**) C*. burnetii* replication between day 1 and day 5 post-infection was measured via RT-PCR. At days 1 and 5 post-infection, bacterial DNA was isolated from two larvae and genomic equivalents (GE) were measured by RT-PCR. The fold replication was determined by dividing the GE from day 5 post-infection by the GE from day 1 post-infection. Shown is the mean of three independent experiments. *p < 0.05. (**d**, **e**) The infection rate of *Galleria mellonella* hemocytes on days 1 and 5 post-infection was determined. Hemocytes from three pooled larvae per infected strain were isolated on days 1 and 5 post-infection and stained for immunofluorescence. (**d**) Representative immunofluorescence micrographs of infected hemocytes are shown. The cytoskeleton was stained with phalloidin (red), *C. burnetii* was stained with an anti-*Coxiella* antibody (green) and the nuclei were stained with DAPI (blue). Scale bars, 10 µm. (**e**) The infection rate of at least 100 hemocytes were scored in three independent experiments. Shown is the mean of three experiments. *p < 0.05; **p < 0.01; ***p < 0.001, n.s. not significant. (**f**) Survival of infected *Galleria mellonella* larvae was analyzed over a period of 8 days. Ten larvae each were infected with 10^6^ bacteria (*C. burnetii* wildtype (WT) or *C. burnetii ΔankG* (ΔAnkG)) per larvae in 20 µl PBS or with a PBS control. Survival was controlled every 24 h. Shown is the mean of three experiments ± SD. ***p < 0.001.

AnkG_NM_ is translocated into the host cell during infection and has anti-apoptotic activity. Whether AnkG is important for the *C. burnetii* infection in vitro and more importantly in vivo is unknown. Recently, the *Galleria mellonella* infection model was used to analyze *C. burnetii* infection in vivo *Galleria mellonella* is susceptible to infection by *C. burnetii* NMI and NMII^24,27,38–40^, suggesting that the pathogenicity of *C. burnetii* is independent of the phase variation in this infection model. Death of the larvae occurred in a dose-dependent manner and depended on a functional T4BSS^38^. Furthermore, this in vivo infection model was successfully used to assess the contribution of the T4BSS effector CvpB/Cig2 during infection^24,27^. Therefore, we used the *Galleria mellonella* model to determine the role of AnkG during an in vivo infection. To assess virulence, a lethal dose of 10^6^ *C. burnetii* NMII wildtype (WT), *C. burnetii* NMII *ΔdotA* (ΔdotA), *C. burnetii* pFlag-AnkF (AnkF) or *C. burnetii* pFlag-AnkG (AnkG) per larvae was injected in the upper right proleg and larval survival was monitored for 7 days. Larvae injected with PBS solution served as a control for the injection stress, the Δ*dotA* mutant for the activity of the T4BSS and AnkF-expressing *C. burnetii* for the overexpression of an effector protein. 90% of the larvae receiving PBS solution survived, whereas 100% of the larvae infected with wildtype bacteria had died by day 7 post-injection. In contrast, the majority (~ 95%) of larvae infected with *C. burnetii ΔdotA* survived (Fig. 7a). This result confirms the importance of the T4BSS for *C. burnetii* virulence. All larvae infected with *C. burnetii* pFlag-AnkG survived till day 6 post-injection while all larvae infected with *C. burnetii* pFlag-AnkF had died by this time point (Fig. 7a). To verify that the observed pro-survival phenotype of larvae infected with *C. burnetii* pFlag-AnkG is due to increased expression of AnkG, we performed qRT-PCR to determine the expression level of AnkG in *C. burnetii* pFlag-AnkG either induced with IPTG or not induced. As shown in Fig. 7b, induction with IPTG resulted in increased expression of AnkG. The expression level decreased over time, but at seven days post-induction it was still significantly higher than in non-induced bacteria. This supports the assumption that the increased translocation of overexpressed AnkG into the host cells mediates improved vitality of the *C. burnetii* infected larvae. To exclude that this phenotype is due to a reduced infection or/and replication ability of the bacteria expressing pFlag-AnkG, we determined the ability of *C. burnetii* to infect and replicate in the larvae by qRT-PCR and immunofluorescence. Only *C. burnetii* Δ*dotA* was unable to replicate in the larvae. In contrast *C. burnetii* WT, Flag-AnkG and Flag-AnkF replicated in the larvae around 100-fold from day 1 to 5 (Fig. 7c). In agreement with this result, we observed that at day 5 post-infection, the majority of hemocytes infected with WT, Flag-AnkG- or Flag-AnkF-expressing *C. burnetii* contained replicative CCVs. This was not the case in hemocytes infected with *C. burnetii* Δ*dotA* (Fig. 7d). These results demonstrate that a functional T4BSS is essential for intracellular replication, as demonstrated before^38^. In addition, these data showed that *C. burnetii* expressing Flag-AnkG replicates in hemocytes. Next, the infection rate of hemocytes was quantified (Fig. 7e). All bacteria exhibited a similar percentage of infection at day 1 post-infection. At day 5 post-infection, the infection rate had increased when infected with WT, Flag-AnkG- or Flag-AnkF-expressing *C. burnetii*, but not when infected with the *C. burnetii ΔdotA* mutant. In the latter case, the rate of infection stayed nearly constant. Thus, *C. burnetii* expressing Flag-AnkG have no defect in the ability to infect hemocytes, to establish a replicative CCV and to spread. We concluded from these data that exogenous AnkG mediates increased survival of *C. burnetii* infected larvae. How AnkG boosts survival of the infected larvae is currently unknown, but it cannot be explained by attenuated bacterial fitness of Flag-AnkG-expressing *C. burnetii*. These data suggest that AnkG might be an important virulence factor. In order to start investigating the role of AnkG for pathogenesis in vivo, we infected larvae with either wild type *C. burnetii* or a Δ*ankG* mutant and monitored their survival over 8 days. Larvae infected with the Δ*ankG* mutant were significantly attenuated in virulence, as demonstrated by improved survival rates (Fig. 7f). However, the underlying reason for this attenuation in virulence will be investigated in future studies. Thus, whether there is a direct or indirect link between the anti-apoptotic activity of AnkG, which might be responsible for the survival of larvae infected with Flag-AnkG-expressing *C. burnetii*, and the attenuation of the Δ*ankG* mutant has to be unraveled.

## Discussion

Comparison of genomes from different *C. burnetii* isolates pointed to a considerable heterogeneity in the repertoire of T4BSS effector proteins^41^. Noticeable variation was detected in the sequences of the *C. burnetii* T4BSS effector CaeA derived from 25 different *C. burnetii* isolates^31^. CaeA was classified in seven different genotypic groups. In the current study, we focused on another effector (AnkG) and analyzed the AnkG nucleotide and amino acid sequences from the same 25 *C. burnetii* isolates in addition to 12 further isolates. These 37 different isolates were divided into three groups according to their respective *ankG* sequences (Fig. 1a,b). To get a better impression of the heterogeneity of the *ankG* sequence, we compared the *ankG* sequences from 57 complete, scaffold and draft genomes of *C. burnetii* strains (Tab. S1). These strains encode nine alleles of the *ankG* gene. Genome groups I, IIb and III^42^ express the wild type sequence of the Nine Mile reference strain (group 1 in this study). Genome group IIa encodes a truncated protein of 92 residues due to a 2 bp frameshift deletion mutation at codons L83N84 and corresponds to AnkG_Soyta_ (group 2 in this study). Genome group IV representatives all contain the Ile to Leu exchange at residue 11 and a 1 bp frameshift insertion mutation at Gly29, leading to a truncated protein with 51 residues and correspond to AnkG_F3_ (group 3 in this study). Three subgroups (designated 3, 3b and 3c in Tab. S1) contain different additional mutations in the remaining 3′ section of *ankG*. Genome groups V and VI both encode a full-length protein with 2 either amino acid substitutions at residues 11 (Ile to Leu) and 72 (Gly to Glu) in genome group V, while genome group VI contains just the mutation at amino acid position 11. There are only three exceptions to this sequence/genome group correlation. The Guyana strain, assigned to genome group I, has a frameshift insertion mutation at codon Gly294, leading to a protein of 327 residues with 33 altered amino acids at its C-terminus. The Cb185 strain, classified as genome group IIa, has the wild type sequence otherwise present in the genome groups I, IIb and III. The strain Cb109, a member of genome group IIb, has a frameshift mutation at residue Asn287, resulting in a protein of 299 residues with 13 altered amino acids at its C-terminus.

Interestingly, only one *C. burnetii* isolate analyzed in this study, encodes AnkG and CaeA, namely *C. burnetii* Nine Mile. Nine isolates encode only the effector protein AnkG and four isolates encode only the effector protein CaeA. Thus, the majority of the strains analyzed encode none or only one of the two anti-apoptotic effector proteins AnkG and CaeA. There seems to be no correlation between the presence of a functional AnkG and/or CaeA effector protein and the geographic distribution of the isolate and its host species. However, this interpretation needs to be taken with caution, as the majority of the isolates were collected from infected ruminants in Germany. Importantly, AnkG and CaeA are not the only *C. burnetii* effector proteins involved in the regulation of host cell viability. So far, two others have also been characterized in more detail: CaeB was shown to inhibit intrinsic apoptosis^23^ and IcaA was described to prevent pyroptosis^21^. However, it can be expected that other members of the so far identified ~ 150 effector proteins might also inhibit host cell death^43^. In line with this assumption, a large-scale transposon mutagenesis coupled to a high-content multi-phenotypic screening in the laboratory of Matteo Bonazzi identified transposon mutants that exhibit a cytotoxic phenotype^7^. This suggests that the genes affected might also contribute to preventing host cell death. Under certain conditions *C. burnetii* also induces apoptosis^44^ or pyroptosis^45^, which might be due to the activity of pro-apoptotic effector proteins that still await identification and characterization. Therefore, the balance between bacterial anti- and pro-apoptotic effector proteins will determine the overall impact of *C. burnetii* on the host cell. In any case, the absence of AnkG and/or CaeA or the expression and translocation of the C-terminal part of AnkG might result in altered pathogenesis of *C. burnetii*. Interestingly, the three different groups of AnkG exhibited different cellular activities (Fig. 2c), although they all bound to the host cell proteins p32 (Fig. 2d) and importin-α1 (Fig. 2e), which was shown to be a prerequisite for AnkG-mediated apoptosis inhibition. Additionally, ectopically expressed AnkG_Soyta_ and AnkG_F3_ are localized within the host cell nucleus, the site of protein activity (Fig. 2f). Furthermore, AnkG_NM_, AnkG_Soyta_ and AnkG_F3_ contain the same first 28 amino acids, which were shown to be necessary and sufficient for anti-apoptotic activity (Fig. 4c, d). The reason for the different cellular activity of the three AnkG variants might be altered protein folding or the inability to interact with themselves or other proteins^46,47^. However, further research is required to identify the underlying reason(s) for the different activities.

To better understand bacterial pathogenesis, appropriate infection models are essential. The *Galleria mellonella* infection model has been recently used to study pathogenic Gram-positive and Gram-negative bacteria^24,27,38,48–52^. Importantly, several of these studies showed a good correlation between the *G. mellonella* and mammalian infection models. For example, the essential role of the type III and type IV secretion systems in virulence was also demonstrated in the *G. mellonella* infection model^38,49,51^. In order to examine the role of specific virulence factors in an in vivo model system, this factor might either be overexpressed or deleted. Here, we used *C. burnetii* NM overexpressing Flag-tagged AnkG^33^. Importantly, *C. burnetii* overexpressing Flag-tagged AnkG showed reduced killing of infected *G. mellonella* (Fig. 7a), although infection and replication were not reduced in comparison to wildtype bacteria (Fig. 7c, d). It can be only speculated how AnkG increased survival of the infected larvae; AnkG might adjust the host immune reaction, possibly by preventing destruction of hemocytes via its anti-apoptotic activity. Hemocytes are important phagocytic immune cells and depletion of circulating hemocytes upon infection correlates with *G. mellonella* mortality^53^. Similarly, the addition of AnkG to the repertoire of *Legionella pneumophila* effector proteins resulted in host tolerance to infection by preventing rapid pathogen-induced apoptosis in dendritic cells^26^.

Taken together, we have demonstrated that AnkG is an anti-apoptotic effector protein, which might mediate host tolerance to a *C. burnetii* infection in the *G. mellonella* model of infection. In addition *C. burnetii* lacking AnkG are attenuated in the *G. mellonella* infection model, indicating that AnkG might be an important virulence factor. Furthermore, we have further narrowed down the anti-apoptotic domain to amino acids 1 to 28 and showed that the effector function is not conserved in all *C. burnetii* isolates. Indeed, our analysis suggests that *C. burnetii* belonging to the genome group IIa and IV do not express a functional AnkG effector protein.

## Methods

### Reagents, cell and bacterial strains

Unless otherwise noted, chemicals were purchased from Sigma Aldrich as described in Schäfer et al.^33^. Complete Protease inhibitor cocktail mixture and Xtreme Gene 9 transfection reagent were purchased from Roche. Staurosporine was bought from Cell Signaling. Cell lines were cultured at 37 °C and 5% CO_2_ in media containing 10% heat-inactivated fetal bovine serum (Biochrom). CHO (Chinese hamster ovary) fibroblasts were grown in minimal essential medium alpha medium (Invitrogen), HEK293T (human embryonic kidney) and MEF cells were maintained in Dulbecco’s modified Eagle’s medium (Invitrogen). *E. coli* strains DH5α and BL21(DE3) were cultivated in Luria–Bertani (LB) broth supplemented with kanamycin or ampicillin if indicated.

### PCR, sequencing and sequence analysis

PCR, sequencing and sequence analysis were done similar to as described earlier in Bisle et al*.*, 2016. Therefore, PCR primers (Table 2) for *ankG* were designed with Primer Select software implemented in Lasergene 9.0 (DNASTAR, Madison, WI). *AnkG* DNA was amplified according to the manufacturer´s protocol using 1 × OptiBuffer (Bioline, London, United Kingdom), 1.4 mM MgCl_2_, 200 μM deoxynucleoside triphosphates (Bioline), 25 pmol of each primer, 1.5 U Bio-X-Act Short DNA polymerase (Bioline), and 5 µl of DNA, in a final volume of 25 µl. The PCR was run with the following thermocycling profile: 5 min at 95 °C; 1 min at 56 °C; 35 cycles, each consisting of 1 min and 30 s at 95 °C, and 30 s at 56 °C, 45 s at 72 °C; and a final elongation step of 10 min at 72 °C. Cycle sequencing was performed using BigDye v1.1 chemistry according to the manufacturer’s instructions (Applied Biosystems) (see Sequencing primers in Table 2), purified with DyeEx 96 plates (Qiagen) and electrophoresed on a 3130XL Genetic Analyzer (Applied Biosystems). Sequence analysis and polymorphism evaluation was performed using Lasergene 9.0 (DNASTAR, Madison, WI) and MEGA5^54^.

**Table 2:** Primers used for *ankG* amplification and sequencing.

Primer designation	Sequence 5′–3′
AnkG_3	GCGCCGCGATCCCGAGATTA (for PCR)
AnkG_4	ACGGGCGAGCAATTACGATTTACG (for PCR)
AnkG_S1	TTCTCATCGTTATCGCATGT (for sequencing)
AnkG_S3	AGGACAGAACCTTTTAGAAC (for sequencing)

### Isolation of genomic *Coxiella burnetii* DNA

Genomic DNAs of 37 paraformaldehyde-fixed or heat-killed *C. burnetii* isolates were prepared according to the manufacturer´s protocol with the illustra bacteria genomic Prep Mini Spin Kit (GE Health Care), with 3 × 10 min incubation at 55 °C in the beginning and a final elution with 30 µl H_2_O as described in Bisle et al., 2016.

### Nuclear fragmentation assay

Nuclear fragmentation assays were performed as described previously^23,31^. In brief, CHO cells were plated on coverslips in 24-well dishes at a density of 2.5 × 10^4^ cells per well. After overnight incubation, cells were transfected with the plasmids indicated. Eighteen hours post-transfection, the cells were incubated with staurosporine (2 mg/ml) for 4 h at 37 °C and 5% CO_2_. The cells were fixed with 4% paraformaldehyde (Alfa Aeser) in PBS (Biochrom) for 20 min at room temperature, permeabilized with ice-cold methanol for 30 s, quenched with 50 mM NH_4_Cl (Roth) in PBS for 15 min at room temperature. The cells were mounted using ProLong Diamond with DAPI (ThermoFisher) to visualize the nucleus.

### Immunoblotting

As described in Schäfer et al.^33^, proteins were separated by SDS/PAGE and transferred to a PVDF membrane (Millipore). The membranes were probed with antibodies directed against GFP (Life Technologies) or HA (Covance Research Products). A chemiluminescence detection system (Thermo Scientific or Millipore) was used to visualize the proteins marked by HRP-conjugated secondary antibodies (Dianova).

### Co-immunoprecipitation

A modified protocol from Schäfer et al.^33^, was used for co-immunoprecipitation. In short, HEK293T cells were transfected with the plasmids indicated. Cells were lysed in lysis buffer (10 mM Tris/HCl [pH 7.5], 150 mM NaCl, 0.5 mM EDTA, 0.5% NP-40) for 30 min on ice and centrifuged for 10 min, 20,000 × g at 4 °C. The supernatants were incubated with GFP-Trap®_A beads (ChromoTek) for 2 h at 4 °C. The beads were washed three times with wash buffer (10 mM Tris/HCl [pH 7.5], 150 mM NaCl, 0.5 mM EDTA). Precipitated proteins were analyzed by immunoblotting.

### Pull-down

Pull-down experiments with *E. coli* BL21(DE3) transformed with a plasmid producing Flag-importin-α1 and lysates of transfected HEK293T cells were performed as described earlier^33^.

### Indirect immunofluorescence

As in Schäfer et al.^33^, cells were seeded on coverslips, washed twice with PBS, fixed with 4% paraformaldehyde (Alfa Aeser) in PBS (Biochrom) for 20 min at room temperature, permeabilized with ice-cold methanol for 30 s, followed by quenching and blocking with 50 mM NH_4_Cl (Roth) in PBS/5% goat serum (Life Technologies) for 30 min at room temperature. The coverslips were incubated with primary antibodies directed against Flag M2 (Sigma Aldrich) and *C. burnetii* diluted in PBS/5% goat serum for 1 h at room temperature, washed three times with PBS and further incubated with secondary Alexa Fluor labelled antibodies Alexa 488 and Alexa 594 (Dianova) diluted in PBS/5% goat serum for 1 h. After three washes with PBS, the cells were mounted using ProLong Diamond with DAPI to visualize cell nuclei and bacterial DNA. Confocal fluorescence microscopy was performed using a Carl Zeiss LSM 700 Laser Scan Confocal Microscope.

### Determination of transcript variations of AnkG

RNA from different AnkG strains was isolated as described in “Quantification of AnkG Level”. For DNase digestion and cDNA synthesis, the RNA was diluted to a 10 ng/µl final concentration. The following strains were used: NMI as member of the group one, 19/34 as member of the second group, and Z3574-1/92 as member of the third group (Table 1). To exclude DNA contamination, a no reverse transcriptase control was performed using 2 µl RNA as template in a PCR reaction with primers 760/761 (*dotA*).

PCR for different *ankG* sections was performed using the DreamTaq DNA polymerase kit (ThermoScientific). Per sample, 25 µl of PCR reagents were used containing 18.8 µl of DNase-free H_2_O, 2.5 µl of DreamTaq Green Buffer, 0.5 µl of dNTPs, 0.5 µl of each Primer (10 µM), and 0.2 µl of polymerase. The amplification was run with the following parameters: 2 min at 95 °C; 30 cycles, each consisting of 30 s, 95 °C, 30 s at 58 °C, and 1 min 72 °C; final elongation of 10 min at 72 °C. Primers were used for the 5´, N-terminus encoding part of the mRNA (a817/a818) and for the full length product (a817 and a822) (Table 3).

**Table 3:** Plasmids used in this study.

Plasmid	Primers*	Reference
pCatch-importin-α1		^59^
pCMV-HA		Clontech
pCMV-HA-AnkG_F3_	329/362	This study
pCMV-HA-AnkG_NM_		^26^
pCMV-HA-AnkG_Soyta_	329/362	This study
pCMV-HA-p32		^26^
pEGFP		Clontech
pEGFP-C3-AcsI		^26^
pEGFP-C3-AnkG_1-28_	1117/a343	This study
pEGFP-AnkG_F3_	25/26	This study
pEGFP-AnkG_F3 1–28_	1117/1111	This study
pEGFP-AnkG_NM_		^26^
pEGFP-AnkG_NM 1–51_	1117/a192	This study
pEGFP-AnkG_NM 1–92_	848/1135	This study
pEGFP-AnkG_Soyta_	25/26	This study
pEGFP-AnkG_Soyta 1–84_	25/1115	This study
pEGFP-AnkG_Soyta 1–86_	25/1116	This study
pcDNA-4TO-Flag		Provided by Alyssa Ingmundson
pcDNA-4TO-Flag-AnkG_Soyta_	54/362	This study
pcDNA-4TO-Flag-AnkG_F3_	54/362	This study
pKM244mod		^33^
pFlag-AnkG_Soyta_	a208/a209	This study
pFlag-AnkG_F3_	a208/a209	This study
pFlag-AnkG_NM_		^33^
pJB-CAT-CyaA		^37^
pJB-CAT-CyaA-AnkG_NM_	a891/a892	This study
pJB-CAT-CyaA-AnkG_Soyta_	a891/a892	This study
pJB-CAT-CyaA-AnkG_F3_	a891/a892	This study

*Primer numbers are as in Table 4.

Amplified *ankG* fragments were analyzed using a 2% agarose (Bio&Sell) gel containing 0.004% midori green (Nippon genetics). The gels were run for 30 min with 120 V and were analyzed using UV light in a gel documentation chamber.

### Axenic cultivation of *C. burnetii* and infection of MEFs

The following experimental procedures were described before^33^. In brief, *C. burnetii* Nine Mile phase II (NMII), transformed with the respective plasmid, was propagated for 6 days in 75 cm^2^ tissue culture flasks at 37 °C and 5% CO_2_, 2.5% O_2_ in 30 ml acidified citrate cysteine medium (ACCM-2), which supports host cell-free (axenic) growth of *C. burnetii*^55^. After 5 days, Isopropyl-β-D-1-thiogalactopyranoside (IPTG) was added to the media where appropriate. The bacteria were pelleted for 30 min at 4.500 × g, 4 °C, resuspended in PBS, pH 7.4, aliquoted and stored at -70 °C for further use. MEFs were seeded 1 day before infection on 10 mm coverslips in 24-well dishes at a density of 2 × 10^4^ cells/well. MEFs were infected with *C. burnetii* at an MOI of 50 in cell culture media containing IPTG. The cells were centrifuged at 250 × g, for 10 min at 20 °C without braking and afterwards incubated at 37 °C and 5% CO_2_ for 48 h.

### Infection of *Galleria mellonella*

*C. burnetii*, *C. burnetii ΔdotA*, *C. burnetii* pFlag-AnkG and *C. burnetii* pFlag-AnkF were grown in 15 ml of axenic ACCM-2 with or without 3 µg/ml chloramphenicol in 25 cm^2^ cell culture filter flasks at 37 °C, 95% CO_2_ and 2.5% O_2_ for 5 days. One day before infection, 2 mM IPTG was added to the *C. burnetii* pFlag-AnkG and *C. burnetii* pFlag-AnkF cultures to induce expression of 3xFlag-AnkG and 3xFlag-AnkF. At the day of infection, *C. burnetii* cultures were pelleted and resuspended in PBS at 5 × 10^4^/µl. *Galleria mellonella* larvae, purchased from TruLarv (UK), were randomized by size into groups of 10 larvae on wood chips. For infection, 20 µl of PBS or *C. burnetii*-containing solutions were injected into the uppermost right leg of the larvae. The larvae were incubated at RT for 7 days with survival monitoring every 24 h. The larvae were regarded as dead when they were not able to move or appeared black.

### Quantification bacterial load/larvae

At day 1 and day 5, two infected *Galleria mellonella* larvae were frozen at -80 °C. On the same day all larvae were disrupted using a BeadRuptor and lysed with Proteinase K overnight. The next day genomic DNA of *C. burnetii* was isolated with the Qiagen DNeasy Blood and Tissue Kit and an RT-PCR was performed to determine the bacterial load. For the fold replication analysis, the genome equivalents of day 5 larvae were compared to the genome equivalents of day 1 larvae. The experiment was performed three times.

### Immunofluorescence of infected hemocytes

Hemocytes from 3 *Galleria mellonella* larvae infected with either *C. burnetii*, *C. burnetii ΔdotA*, *C. burnetii pFlag-AnkG* or *C. burnetii pFlag-AnkF* were collected at 1 day and 5 days post infection^38^. Cells were seeded and centrifuged on poly-l-lysine-coated coverslips, washed twice with PBS, fixed with 4% paraformaldehyde (Alfa Aeser) in PBS (Biochrom) for 20 min at room temperature, permeabilized with 0.1% Triton- X 100, followed by quenching and blocking with 50 mM NH_4_Cl (Roth) in PBS/5% goat serum (Life Technologies) for 30 min at room temperature. The coverslips were incubated with primary antibodies directed against *C. burnetii* and actin (phalloidin labeled with Alexa Fluor 674) diluted in PBS/5% goat serum for 20 min at room temperature, washed three times with PBS and further incubated with secondary Alexa Fluor labelled antibodies Alexa 488 diluted in PBS/5% goat serum for 20 min. After three washes with PBS, the cells were mounted using ProLong Diamond with DAPI to visualize cell nuclei and bacterial DNA. Confocal fluorescence microscopy was performed using a Carl Zeiss LSM 700 Laser Scan Confocal Microscope. In Z-Stack images with 1 µm distance 100 randomized cells were counted and determined whether they were infected or not. The experiment was performed three times.

### Targeted DotA deletion in *C. burnetii* NMII

Targeted gene deletion of the NMII gene *dotA* was performed as described previously by homologous recombination with a suicide plasmid^56^. Briefly, 1 × 10^10^ electrocompetent *C. burnetii* NMII were transformed with the plasmid harboring 2 kb 5′ and 3′ flanking region of genomic *dotA* CDS flanking a *P*_*Com1*_*-CAT* cassette (BioRad Gene Pulser XCell, 18,000 V/cm^2^, 500 Ω, 25 µF in 0.1 cm gap cuvettes). Subsequently, transformed NMII were cultivated axenically in 6 ml 1xACCM-2 with 3 µg/ml chloramphenicol and/or 350 µg/ml kanamycin at 37 °C and 2.5% O_2_ atmosphere in T25 flasks for 6 days. Afterwards, cultures were passaged every 7 d for 3 weeks in 6 ml medium supplemented with chloramphenicol (CM) and kanamycin in T25 flasks. Identification of plasmid co-integrants was performed by colony PCR with primers as proposed^56^. Co-integrants were then subcultured in 3 ml medium supplemented with CM and 1% sucrose in 6-well plates for 4 d, followed by two passages for 6 d in 6 ml medium supplemented with CM in T25 flasks until late stationary phase. Identification of *dotA* knock-out (*ΔdotA*) strains was performed by colony PCR with specific primers^56^. Clonal isolation of *ΔdotA* strains was performed through plating on semi-solid agar plates.

### Translocation assay

AnkG_NM_, AnkG_Soyta_ and AnkG_F3_ were cloned into the pJB-CAT-CyaA plasmid. *C. burnetii* grown in ACCM-D medium were electroporated with 10 µg pJB-CAT-Cya-AnkG_Soyta_, pJB-CAT-Cya-AnkG_NM_ or pJB-CAT-Cya-AnkG_F3_. Transformants were selected by culturing the bacteria in ACCM-D medium containing 3 µg/ml chloramphenicol for 5 days, plated onto ACCM-D/0.25% agarose plates supplemented with 3 µg/ml chloramphenicol. Single clones were picked after 7–10 days as previously described^56^. CHO cells were infected with the different *C. burnetii* mutants in a 24 well plate at an MOI of 200 for 3 days at 37 °C and 5% CO_2_. The translocation assay was performed by measuring the concentration of cAMP in cell lysates using the cAMP enzyme immunoassay (GE Healthcare) as previously described^19^. The threshold was set as 2.5-fold more cAMP than the controls (uninfected cells and cells infected with *C. burnetii* expressing CyaA alone)^25^.

### Plasmid construction

Plasmids used in this study are listed in Table 3. The AnkG_Soyta_ and AnkG_F3_ genes were amplified from *C. burnetii* Soyta or F3 genomic DNA by standard PCR using the primers listed in Table 4. The resulting PCR product was restricted with AscI, followed by ligation with likewise-restricted pEGFP-C3-AscI.

**Table 4:** Primers used in this study.

Primer no	Sequence*	Site
25	5′-AAGGCGCGCCAAGTAGACGTGAGACTCCC-3′	AscI
26	5′-AAGGCGCGCCTCACCGAGGACTAGACAG-3′	AscI
54	5′-CCGGATCCATGAGTAGACGTGAGACTCC-3′	BamHI
65	5′-CCGAGACTCCCACTAGCACAAT-3′	–
66	5′-CCCTCTACTCGAAAATGGCGTA-3′	–
329	5′-CCAAGATCTCTATGAGTAGACGTGAGACTCC-3′	BglII
362	5′-CCAATTGCGGCCGCTCACCGAGGACTAGACAGA-3′	NotI
737	5′-GCGCAATACGCTCAATCACA-3′	–
738	5′-CCATGGCCCCAATTCTCTT-3′	–
760	GCGCAATACGCTCAATCACA	–
761	CCATGGCCCCAATTCTCTT	–
848	5′-CCCCTCGAGCCAGTAGACGTGAGACTCCCA-3′	XhoI
1111	5′-CGGGGTACCTCATTTTCGGCTCAATCTCCTTCT-3′	KpnI
1115	5′-CGGGGTACCTCAGGCGGTACGTAGGCCCC-3′	KpnI
1116	5′-CGGGGTACCTCAACGTAGGCCCCATTTCGCC-3′	KpnI
1117	5′-CCGCATATGCCAAGTACGCCCC-3′	NdeI
1135	5′-CGGGGTACCTCACTGGAAATCCGTCTTTGGCG-3′	KpnI
a192	5′-CGCGGATCCTCAAAACGATAAATCGAATACAGTAAA-3′	BamHI
a208	5′-TTCGAGCTCGGTACCATGGACTACAAAGACCATGACGG-3´	KpnI
a209	5′-GCATCTAGAGGTACCTCACCGAGGACTAGACAGA-3′	KpnI
a343	5′-CGCGGATCCTCATTTTCGGCTCAATCTCCTTC-3′	BamHI
a808	5′-GGCTGGCGCTTACAAAGAGATAGTC-3′	–
a817	5′-GAGTAGACGTGAGACTCCCACTAG-3′	–
a818	5′-CGATAAATCGAATACAGTAAATATCGAATTCTTTGC-3′	–
a822	5′-CCGAGGACTAGACAGACAAGAGAGAG-3′	–
a891	5′-CCGAAGCGGTGTCGACATGAGTAGACGTGAGACTCC-3′	SalI
a892	5′-CCCATGCCTCAGTCGACTCACCGAGGACTAGACAGAC-3′	SalI
*Underlining indicates the location of the restriction site		

For creation of the constructs pJB-CAT-Cya-AnkG_NM_, pJB-CAT-Cya-AnkG_Soyta_ and pJB-CAT-Cya-AnkG_F3_, the genes were amplified from pEGFP-AnkG_NM_, pEGFP-AnkG_Soyta_ and pEGFP-AnkG_F3_ using the primers listed in Table 4, restricted with SalI, and ligated with likewise-restricted pJB-CAT-Cya.

For creation of the constructs pCMV-HA-AnkG_Soyta_ and pCMV-HA-AnkG_F3_, the genes were amplified from pEGFP-AnkG_Soyta_ and pEGFP-AnkG_F3_ using the primers listed in Table 4, restricted with BglII and NotI, and ligated with likewise-restricted pCMV-HA.

For cloning of the constructs pEGFP-AnkG_NM 1–51_, pEGFP-AnkG_NM 1–92_, pEGFP-AnkG_F3 1–28_, pEGFP-AnkG_Soyta 1–84_ and pEGFP-AnkG_Soyta 1–86_, the genes were amplified from pEGFP-AnkG_NM_, pEGFP-AnkG_Soyta_, pEGFP-AnkG_F3_, using the primers listed in Table 4, restricted as indicated, and ligated with likewise-restricted pEGFP-C1, C2, C3 or pEGFP-C3-AscI.

For creation of the constructs pcDNA–4TO–Flag–AnkG_Soyta_, and pcDNA–4TO–Flag-AnkG_F3_, the genes were amplified from pCMV–HA–AnkG_Soyta_ and pCMV–HA–AnkG_F3_ using the primers listed in Table 4, restricted with BamHI and NotI, and ligated with likewise-restricted pcDNA–4TO–Flag.

For cloning of the constructs pFlag–AnkG_Soyta_ and pFlag–AnkG_F3_, the genes were amplified from pcDNA–4TO–Flag–AnkG_Soyta_ or pcDNA–4TO–Flag–AnkG_F3_ using the primers listed in Table 4, restricted with KpnI, and ligated with likewise-restricted pKM244mod.

The construct pEGFP-AnkG_1-28_ was produced using the primers in Table 4. As template pEGFP-AnkG_NM_ was used and AnkG_1-28_ coupled to GFP was amplified and ligated in a pEGFP backbone lacking GFP. The restriction enzymes NdeI and BamHI were used for directed cloning.

For construction of pJC-CAT::*ankG*-5′3′-*lysCA*, the 5′ and 3′ regions of *ankG* were amplified by PCR from NMI genomic DNA using the specific primer sets (5′- CGGTACCCGGGGATCCGATCGATTACTGCAGAGAAGC and 3′- CACCCATATGCGACGCGAGCGTCGAGAGAATATCCTTATTTTTGATGTC) and (5′- CGTCGCATATGGGTGCGCATGTACGTCAAAAGATGAGGGGTGCTAATG and 3′- GAACCTGTTTGTCGACCAATAACGCTAAGAATAATAATATAG), respectively. The 5′ and 3′ PCR products were cloned into BamHI/SalI-digested pJC-CAT by In-Fusion, resulting in formation of an internal NdeI site between the 5′ and 3′ fragments and creation of pJC-CAT::*ankG*-5′3′. The *1169*^*P*^*-lysCA* cassette was amplified from pJC-CAT::*1169*^*P*^*-lysCA*^57^ by PCR with specific primers (5′-GCTCGCGTCGCATATGGAGCTCGGTACCCGGGGATCC and 3′-CATGCGCACCCATATGGATTAATTAGAGAACCTGTTTGTCGAC) and cloned by In-Fusion into NdeI-digested pJC-CAT::*ankG*-5′3′ to create pJC-CAT::*ankG*-5′3′*-lysCA*.

### Generation of a* C. burnetii ΔankG* mutant

*C. burnetii* Nine Mile phase II were electroporated with 10 µg pJC-CAT::*ankG*-5´3´-*lysCA* as previously described^58^. Co-integrants were selected by culturing the bacteria in ACCM-D media lacking lysine, but containing 2% sucrose for 4 days as previously described^57^. Surviving transformants were expanded in ACCM-D media lacking lysine for 7 days. After spreading the diluted culture on 0.25% ACCM-D agarose without lysine clonal Δ*ankG* mutants were picked after 7 days of culture. The picked clones were expanded in ACCM-D media without lysine.

### Quantification of the* ankG* expression level

RNA was isolated using the TriFast Reagent (VWR) according to the manufacturer’s instructions and treated with DNase (RNase-Free DNase Set, Qiagen). Therefore, 1,000 ng RNA were digested using 2 µl RDD Buffer and 1 µl DNase (ad 20 µl). cDNA synthesis was performed with SuperScriptII reverse transcriptase (Thermo Scientific) as recommend by the manufacturer using specific primers for *ankG* and *dotA*. Subsequent quantification by qRT-PCR was performed using the SybrGreen qPCR Mix (Thermo Scientific) with primers for *ankG* (65/66) and *dotA* (737/738) for normalization (Table 4).

### Statistical analysis

An unpaired Student´s t-test was used for statistical analysis.

## Supplementary information

Supplementary information.
