# Supplementary material for: The anti-apoptotic Coxiella burnetii effector protein AnkG is a strain specific virulence factor
Source: Sci Rep. 2020 Sep 21;10:15396. doi: 10.1038/s41598-020-72340-9 (PMC7506536; doi:10.1038/s41598-020-72340-9)
Supplement: Supplementary file 1 — Supplementary information. [file 41598_2020_72340_MOESM1_ESM.pdf]

SUPPLEMENTAL INFORMATION

The anti-apoptotic *Coxiella burnetii* effector protein AnkG is a strain specific virulence factor

Walter Schäfer[1], Teresa Schmidt[1], Arne Cordsmeier[1], Vítor Borges[2], Paul A. Beare[3], Julian Pechstein[1], Jan Schulze-Luehrmann[1], Jonas Holzinger[1], Nicole Wagner[4], Christian Berens[4], Carsten Heydel[5], João Paulo Gomes[2] and Anja Lührmann[1,*]

[1]Mikrobiologisches Institut, Universitätsklinikum Erlangen, Friedrich-Alexander Universität Erlangen-Nürnberg, Erlangen, Germany

[2]National Institute of Health, Department of Infectious Diseases, Lisbon, Portugal

[3]Coxiella Pathogenesis Section, Laboratory of Bacteriology, Rocky Mountain Laboratories, National Institute of Allergy and Infectious Diseases, National Institutes of Health, Hamilton, Montana, United States of America

[4]Institut für molekulare Pathogenese, Friedrich-Loeffler-Institut, 07743 Jena, Germany

[5]Institut für Hygiene und Infektionskrankheiten der Tiere, Justus Liebig Universität Gießen, Frankfurter Straße 85-89, 35392 Gießen, Germany

Correspondence:

PD Dr. Anja Lührmann, Mikrobiologisches Institut – Klinische Mikrobiologie, Immunologie und Hygiene Universitätsklinikum Erlangen, Friedrich-Alexander Universität Erlangen-Nürnberg, Wasserturmstraße 3/5, 91054 Erlangen, Germany; Email: anja.luehrmann@uk-erlangen.de

Table S1: Analysis of *ankG* in 57 *C. burnetii* strains

The genome assemblies of *Coxiella burnetii* strains, which had been uploaded to the NCBI Genome website (https://www.ncbi.nlm.nih.gov/genome/), at the complete genome, chromosome, scaffold and contig levels and for which information on their genome group classification was known [13], were downloaded, discarding multiple strain entries or passage variants. Their *ankG* sequences were identified by BLAST analysis with the *ankG* coding sequence from the RSA493 Nine Mile strain serving as reference. The AnkG groups were classified according to protein length with group 1 representing the full-length protein. Group 2 encodes a truncated protein of 92 residues due to a 2bp frameshift deletion mutation at codons L83N84 while group 3 representatives all contain an Ile to Leu exchange at residue 11 and a 1bp frameshift insertion mutation at Gly29, leading to a truncated protein with 51 residues. The subgroups 1b, 1c, 3b and 3c contain additional mutations that do not affect AnkG protein length. The strain Cb109, the sole member of AnkG group 4, has a frameshift mutation at residue Asn287, resulting in a protein of 299 residues with 13 altered amino acids at its C-terminus. Finally, the Guyana strain, representing AnkG group 5, has a frameshift insertion mutation at codon Gly294, leading to a protein of 327 residues with 33 altered amino acids at its C-terminus.

**Genome Group**	**Strain**	**AnkG group**	**Accession #**	**Sequence Reference**
**I**	RSA493 (NM-I)	1	AE016828	[1]
	RSA315 (Turkey)	1	NOLO00000000	[2]
	RSA435 (Dyer)	1	NOLQ00000000	[2]
	RSA270 (Ohio314)	1	NOLT00000000	[3]
	RSA329 (California33)	1	NOLV00000000	[3]
	RSA350 (California16)	1	NOLU00000000	[3]
	RSA514 (NM-Crazy)	1	NOVG00000000	[4]
	Cb_C2	1	CCAJ010000000	[5]
	Cb175_Guyana	5	HG825990	[6]
**IIa**	RSA331 (Henzerling)	2	CP000890, CP014559	[7, 8]
	Heizberg	2	CP014561	[8]
	RSA461, M44_Clone1	2	NOVI00000000	[4]
	Cb185	1	CBTH01000000	[9]
**IIb**	CbCVIC1	1	CP014549	[8]
	Z3055	1	LK937696	[10]
	NL-Limburg	1	JZWL01	[11]

	NL3262	1	CP013667	[12]
	NLhu3345937	1	CP014354	[12]
	602 (14160-002)	1	CP014836	[8]
	42785537	1	CP014548	[8]
	EV-Cb_C13	1	CCAM010000000	[5]
	Q540	1	PPFP01000000	[13]
	Cb_D2 (DSTL_2)	1	RQJT01000000	[13]
	Cb_D8 (DSTL_8)	1	RQJS01000000	[13]
	Cb_D10 (DSTL_10)	1	RQJR01000000	[13]
	Cb109	4	AKYP01000000	[14]
**III**	Idaho Goat_Q195	1	NOLR00000000	[3]
	2574	1	CP014555	[8]
	601 (14160-001)	1	CP014551	[8]
	18430	1	CP014557	[8]
	701CbB1	1	CP014553	[8]
	Cb_B1	1	CCAH010000000	[5]
	Cb_B18	1	CCAI010000000	[5]
	EV-Cb_BK18	1	CCAL010000000	[5]
	Q532	1	PPFQ01000000	[13]
	Q545	1	PPFO01000000	[13]
	Cb_D1 (DSTL_1R)	1	RQJU01000000	[13]
	Q556		PPFN01000000	[13]
	Q559	1	PPFM01000000	[13]
**IV**	Schperling	3	CP014563	[8]
	Cbu_K154	3	CP001020	[15]
	'MSU Goat Q177 (Priscilla)	3	CP018150	[16]
	Leningrad-2	3c	PDLP00000000	[17]
	Namibia	3b	CP007555	[18]
	AuQ01 (Arandale)	3c	JPVV01000000	[19]
	Cb196_SaudiArabia	3	CCXO01000000	[20]
	Q321	3	AAYJ01000000	[21]
	Cb_O184	3	CCAK010000000	[5]
	Cb171_QLYMPHOMA	3	CDBG01000000	[22]
**V**	Cbu G_Q212	1c	CP001019	[14]
	Scurry S_Q217	1c	CP014565	[8]
	Ko_Q229	1c	NOLP00000000	[2]
	Dog Utad	1c	CCNR00000000	[23]
**VI**	Dugway 5J108-111, 7D77-80, 7E65-68	1b	CP000733; NOLN00000000, NOLM00000000	[14, 24]

Supplemental References

1. Seshadri, R. *et al.*. Complete genome sequence of the Q-fever pathogen *Coxiella burnetii*. *Proc Natl Acad Sci USA* **100**, 5455-5460 (2003).
2. Beare, P.A., Jeffrey, B.M., Martens, C.A. & Heinzen, R.A. Draft genome sequences of three *Coxiella burnetii* strains isolated from Q fever patients. *Genome Announc* **5**, e00986-17 (2017a).
3. Beare, P.A., Jeffrey, B.M., Martens, C.A., Pearson, T. & Heinzen, R.A. Draft genome sequences of historical strains of *Coxiella burnetii* isolated from cow's milk and a goat placenta. *Genome Announc* **5**, e00985-17 (2017b).
4. Beare, P.A., Jeffrey, B.M., Long, C.M., Martens, C.M. & Heinzen, R.A. Genetic mechanisms of *Coxiella burnetii* lipopolysaccharide phase variation. *PLoS Pathog* **14**, e1006922 (2018).
5. Sidi-Boumedine, K. *et al.* Draft genome sequences of six ruminant *Coxiella burnetii* isolates of European origin. *Genome Announc* **2**, e00285-14 (2014).
6. D'Amato F. *et al.* Loss of TSS1 in hypervirulent *Coxiella burnetii* 175, the causative agent of Q fever in French Guiana. *Comp Immunol Microbiol Infect Dis* **41**, 35-41 (2015).
7. Beare, P.A. *et al.* Genetic diversity of the Q fever agent, *Coxiella burnetii,* assessed by microarray-based whole-genome comparisons. *J Bacteriol* **188**, 2309-2324 (2006).
8. Kuley, R. *et al.* Genome plasticity and polymorphisms in critical genes correlate with increased virulence of Dutch outbreak-related *Coxiella burnetii* strains. *Front Microbiol* **8**, 1526 (2017).
9. Million, M. *et al.* Reevaluation of the risk of fetal death and malformation after Q Fever. *Clin Infect Dis* **59**, 256-260 (2014).
10. D'Amato, F. *et al.* The genome of *Coxiella burnetii* Z3055, a clone linked to the Netherlands Q fever outbreaks, provides evidence for the role of drift in the emergence of epidemic clones. *Comp Immunol Microbiol Infect Dis* **37**, 281-288 (2014a).
11. Hammerl, J. A. *et al.* First draft genome sequence of a human *Coxiella burnetii* isolate, originating from the largest Q fever outbreak ever reported, the Netherlands, 2007 to 2010. *Genome Announc* **3**, e00445-15 (2015).
12. Kuley, R. *et al.* First complete genome sequence of the Dutch veterinary *Coxiella burnetii* strain NL3262, originating from the largest global Q fever outbreak, and draft genome sequence of its epidemiologically linked chronic human isolate NLhu3345937. *Genome Announc* **4**, e00245-16 (2016).
13. Hemsley, C. M. *et al.* Extensive genome analysis of *Coxiella burnetii* reveals limited evolution within genomic groups. *BMC genomics* **20**, 441 (2019).
14. Rouli, L. *et al.* Genome sequence of *Coxiella burnetii* 109, a doxycycline-resistant clinical isolate. *J Bacteriol* **194**, 6939 (2012).
15. Beare, P. A. *et al.* Comparative genomics reveal extensive transposon-mediated genomic plasticity and diversity among potential effector proteins within the genus *Coxiella*. *Infect Immun* **77**, 642-656 (2009).
16. Walter, M. C., Frangoulidis, D., Seshadri, R. & Samuel, J. E. Resequencing and curated structural and functional annotation of the *Coxiella burnetii* reference genomes (2016); unpublished.
17. Freylikhman, O. A. *et al.* Draft genome sequences of historical strain of *Coxiella burnetii is*olated from human blood of patient with acute Q fever in Russia (2017); unpublished.

18. Walter, M. C. *et al.* Genome sequence of *Coxiella burnetii* strain Namibia. *Stand in Genomic Sci* **9**, 22 (2014a).
19. D´Amato, F., Robert, C., Azhar, E. I., Fournier, P. E. & Raoult, D. Draft genome sequence of *Coxiella burnetii* strain Cb196, an agent of endocarditis in Saudia Arabia. *Genome Announc* **2**, e01180-14 (2014b).
20. Walter, M. C., Vincent, G. A., Stenos, J., Graves, S. & Frangoulidis, D. Genome Sequence of *Coxiella burnetii* strain AuQ01 (Arandale) from an Australian patient with acute Q fever. *Genome Announc* **2**, e00964-14 (2014b).
21. Seshadri, R. & Samuel, J. E. Genome sequencing of phylogenetically and phenotypically diverse *Coxiella burnetii* isolates (2007); unpublished.
22. Urmite Genomes. *Coxiella burnetii* RSA 493 strain Cb171_QLYMPHOMA, whole genome shotgun sequencing project. Direct Submission (2014); unpublished.
23. D'Amato, F. *et al.* Draft genome sequence of *Coxiella burnetii* Dog Utad, a strain isolated from a dog-related outbreak of Q fever. *New Microbes New Infect* **2**, 136-137 (2014b).
24. Beare, P.A., Jeffrey, B. M., Martens, C. A. & Heinzen, R. A. Draft genome sequences of the avirulent *Coxiella burnetii* Dugway 7D77-80 and Dugway 7E65-68 strains isolated from rodents in Dugway, Utah. *Genome Announc* **5**, e00984-17 (2017c).
